# SCIM: universal single-cell matching with unpaired feature sets

**DOI:** 10.1093/bioinformatics/btaa843

**Published:** 2020-12-29

**Authors:** Stefan G Stark, Joanna Ficek, Francesco Locatello, Ximena Bonilla, Stéphane Chevrier, Franziska Singer, Tumor Profiler Consortium, Rudolf Aebersold, Faisal S Al-Quaddoomi, Jonas Albinus, Ilaria Alborelli, Sonali Andani, Per-Olof Attinger, Marina Bacac, Daniel Baumhoer, Beatrice Beck-Schimmer, Niko Beerenwinkel, Christian Beisel, Lara Bernasconi, Anne Bertolini, Bernd Bodenmiller, Ximena Bonilla, Ruben Casanova, Stéphane Chevrier, Natalia Chicherova, Maya D'Costa, Esther Danenberg, Natalie Davidson, Monica-Andreea Dră gan, Reinhard Dummer, Stefanie Engler, Martin Erkens, Katja Eschbach, Cinzia Esposito, André Fedier, Pedro Ferreira, Joanna Ficek, Anja L Frei, Bruno Frey, Sandra Goetze, Linda Grob, Gabriele Gut, Detlef Günther, Martina Haberecker, Pirmin Haeuptle, Viola Heinzelmann-Schwarz, Sylvia Herter, Rene Holtackers, Tamara Huesser, Anja Irmisch, Francis Jacob, Andrea Jacobs, Tim M Jaeger, Katharina Jahn, Alva R James, Philip M Jermann, André Kahles, Abdullah Kahraman, Viktor H Koelzer, Werner Kuebler, Jack Kuipers, Christian P Kunze, Christian Kurzeder, Kjong-Van Lehmann, Mitchell Levesque, Sebastian Lugert, Gerd Maass, Markus Manz, Philipp Markolin, Julien Mena, Ulrike Menzel, Julian M Metzler, Nicola Miglino, Emanuela S Milani, Holger Moch, Simone Muenst, Riccardo Murri, Charlotte KY Ng, Stefan Nicolet, Marta Nowak, Patrick GA Pedrioli, Lucas Pelkmans, Salvatore Piscuoglio, Michael Prummer, Mathilde Ritter, Christian Rommel, María L Rosano-González, Gunnar Rätsch, Natascha Santacroce, Jacobo Sarabia del Castillo, Ramona Schlenker, Petra C Schwalie, Severin Schwan, Tobias Schär, Gabriela Senti, Franziska Singer, Sujana Sivapatham, Berend Snijder, Bettina Sobottka, Vipin T Sreedharan, Stefan Stark, Daniel J Stekhoven, Alexandre PA Theocharides, Tinu M Thomas, Markus Tolnay, Vinko Tosevski, Nora C Toussaint, Mustafa A Tuncel, Marina Tusup, Audrey Van Drogen, Marcus Vetter, Tatjana Vlajnic, Sandra Weber, Walter P Weber, Rebekka Wegmann, Michael Weller, Fabian Wendt, Norbert Wey, Andreas Wicki, Bernd Wollscheid, Shuqing Yu, Johanna Ziegler, Marc Zimmermann, Martin Zoche, Gregor Zuend, Gunnar Rätsch, Kjong-Van Lehmann

**Affiliations:** Department of Computer Science, ETH Zürich, 8092 Zürich, Switzerland; Swiss Institute of Bioinformatics, Quartier Sorge Bâtiment Amphipôle, 1015 Lausanne, Switzerland; Life Science Zurich Graduate School, PhD Program Molecular & Translational Biomedicine, 8057 Zürich, Switzerland; Department of Computer Science, ETH Zürich, 8092 Zürich, Switzerland; Swiss Institute of Bioinformatics, Quartier Sorge Bâtiment Amphipôle, 1015 Lausanne, Switzerland; Life Science Zurich Graduate School, PhD Program Molecular & Translational Biomedicine, 8057 Zürich, Switzerland; Max Planck Institute for Intelligent Systems, Empirical Inference Department, 72076 Tübingen, Germany; Department of Computer Science, ETH Zürich, 8092 Zürich, Switzerland; Center for Learning Systems, ETH Zürich, 8092 Zürich, Switzerland; Department of Quantitative Biomedicine, University of Zürich, 8057 Zürich, Switzerland; Department of Computer Science, ETH Zürich, 8092 Zürich, Switzerland; Swiss Institute of Bioinformatics, Quartier Sorge Bâtiment Amphipôle, 1015 Lausanne, Switzerland; Life Science Zurich Graduate School, PhD Program Molecular & Translational Biomedicine, 8057 Zürich, Switzerland; University Hospital Zürich, 8091 Zürich, Switzerl; Swiss Institute of Bioinformatics, Quartier Sorge Bâtiment Amphipôle, 1015 Lausanne, Switzerland; University Hospital Zürich, 8091 Zürich Switzerland; ETH Zurich, Department of Biology, Wolfgang-Pauli-Strasse 27, 8093 Zurich, Switzerland; ETH Zurich, NEXUS Personalized Health Technologies, John-von-Neumann-Weg 9, 8093 Zurich, Switzerland; Swiss Institute of Bioinformatics, Zurich, Switzerland; ETH Zurich, Department of Health Sciences and Technology, Otto-Stern-Weg 3, 8093 Zurich, Switzerland; University Hospital Basel, Institute of Medical Genetics and Pathology, Schönbeinstrasse 40, 4031 Basel, Switzerland; ETH Zurich, Department of Biology, Wolfgang-Pauli-Strasse 27, 8093 Zurich, Switzerland; ETH Zurich, Department of Computer Science, Institute of Machine Learning, Universitätstrasse 6, 8092 Zurich, Switzerland; Swiss Institute of Bioinformatics, Zurich, Switzerland; University Hospital Zurich, Biomedical Informatics, Schmelzbergstrasse 26, 8006 Zurich, Switzerland; ETH Zurich, Department of Biology, Wolfgang-Pauli-Strasse 27, 8093 Zurich, Switzerland; F. Hoffmann-La Roche Ltd, Grenzacherstrasse 124, 4070 Basel, Switzerland; F. Hoffmann-La Roche Ltd, Grenzacherstrasse 124, 4070 Basel, Switzerland; Roche Pharmaceutical Research and Early Development, Roche Innovation Center Zurich, Wagistrasse 10, 8952 Schlieren, Switzerland; University Hospital Basel, Institute of Medical Genetics and Pathology, Schönbeinstrasse 40, 4031 Basel, Switzerland; University of Zurich, VP Medicine, Künstlergasse 15, 8001 Zurich, Switzerland; ETH Zurich, Department of Biosystems Science and Engineering, Mattenstrasse 26, 4058 Basel, Switzerland; ETH Zurich, Department of Biosystems Science and Engineering, Mattenstrasse 26, 4058 Basel, Switzerland; University Hospital Zurich, Clinical Trials Center, Rämistrasse 100, 8091 Zurich, Switzerland; ETH Zurich, NEXUS Personalized Health Technologies, John-von-Neumann-Weg 9, 8093 Zurich, Switzerland; Swiss Institute of Bioinformatics, Zurich, Switzerland; University of Zurich, Department of Quantitative Biomedicine, Winterthurerstrasse 190, 8057 Zurich, Switzerland; ETH Zurich, Department of Biology, Wolfgang-Pauli-Strasse 27, 8093 Zurich, Switzerland; ETH Zurich, Department of Computer Science, Institute of Machine Learning, Universitätstrasse 6, 8092 Zurich, Switzerland; Swiss Institute of Bioinformatics, Zurich, Switzerland; University Hospital Zurich, Biomedical Informatics, Schmelzbergstrasse 26, 8006 Zurich, Switzerland; University of Zurich, Department of Quantitative Biomedicine, Winterthurerstrasse 190, 8057 Zurich, Switzerland; University of Zurich, Department of Quantitative Biomedicine, Winterthurerstrasse 190, 8057 Zurich, Switzerland; ETH Zurich, NEXUS Personalized Health Technologies, John-von-Neumann-Weg 9, 8093 Zurich, Switzerland; Swiss Institute of Bioinformatics, Zurich, Switzerland; F. Hoffmann-La Roche Ltd, Grenzacherstrasse 124, 4070 Basel, Switzerland; University of Zurich, Institute of Molecular Life Sciences, Winterthurerstrasse 190, 8057 Zurich, Switzerland; ETH Zurich, Department of Biology, Wolfgang-Pauli-Strasse 27, 8093 Zurich, Switzerland; ETH Zurich, Department of Computer Science, Institute of Machine Learning, Universitätstrasse 6, 8092 Zurich, Switzerland; Swiss Institute of Bioinformatics, Zurich, Switzerland; University Hospital Zurich, Biomedical Informatics, Schmelzbergstrasse 26, 8006 Zurich, Switzerland; ETH Zurich, Department of Biosystems Science and Engineering, Mattenstrasse 26, 4058 Basel, Switzerland; ETH Zurich, Department of Biology, Wolfgang-Pauli-Strasse 27, 8093 Zurich, Switzerland; ETH Zurich, Department of Health Sciences and Technology, Otto-Stern-Weg 3, 8093 Zurich, Switzerland; University of Zurich, Department of Quantitative Biomedicine, Winterthurerstrasse 190, 8057 Zurich, Switzerland; Roche Pharmaceutical Research and Early Development, Roche Innovation Center Basel, Grenzacherstrasse 124, 4070 Basel, Switzerland; ETH Zurich, Department of Biosystems Science and Engineering, Mattenstrasse 26, 4058 Basel, Switzerland; University of Zurich, Institute of Molecular Life Sciences, Winterthurerstrasse 190, 8057 Zurich, Switzerland; University Hospital Basel and University of Basel, Department of Biomedicine, Hebelstrasse 20, 4031 Basel, Switzerland; ETH Zurich, Department of Biosystems Science and Engineering, Mattenstrasse 26, 4058 Basel, Switzerland; ETH Zurich, Department of Biology, Wolfgang-Pauli-Strasse 27, 8093 Zurich, Switzerland; ETH Zurich, Department of Computer Science, Institute of Machine Learning, Universitätstrasse 6, 8092 Zurich, Switzerland; Swiss Institute of Bioinformatics, Zurich, Switzerland; University Hospital Zurich, Biomedical Informatics, Schmelzbergstrasse 26, 8006 Zurich, Switzerland; ETH Zurich, Department of Biology, Wolfgang-Pauli-Strasse 27, 8093 Zurich, Switzerland; F. Hoffmann-La Roche Ltd, Grenzacherstrasse 124, 4070 Basel, Switzerland; Roche Diagnostics GmbH, Nonnenwald 2, 82377 Penzberg, Germany; ETH Zurich, Department of Health Sciences and Technology, Otto-Stern-Weg 3, 8093 Zurich, Switzerland; ETH Zurich, NEXUS Personalized Health Technologies, John-von-Neumann-Weg 9, 8093 Zurich, Switzerland; Swiss Institute of Bioinformatics, Zurich, Switzerland; University of Zurich, Institute of Molecular Life Sciences, Winterthurerstrasse 190, 8057 Zurich, Switzerland; ETH Zurich, Department of Chemistry and Applied Biosciences, Vladimir-Prelog-Weg 1-5/10, 8093 Zurich, Switzerland; ETH Zurich, Department of Biology, Wolfgang-Pauli-Strasse 27, 8093 Zurich, Switzerland; F. Hoffmann-La Roche Ltd, Grenzacherstrasse 124, 4070 Basel, Switzerland; Cantonal Hospital Baselland, Medical University Clinic, Rheinstrasse 26, 4410 Liestal, Switzerland; University Hospital Basel and University of Basel, Department of Biomedicine, Hebelstrasse 20, 4031 Basel, Switzerland; University Hospital Basel, Gynecological Cancer Center, Spitalstrasse 21, 4031 Basel, Switzerland; Roche Pharmaceutical Research and Early Development, Roche Innovation Center Zurich, Wagistrasse 10, 8952 Schlieren, Switzerland; University of Zurich, Institute of Molecular Life Sciences, Winterthurerstrasse 190, 8057 Zurich, Switzerland; Roche Pharmaceutical Research and Early Development, Roche Innovation Center Zurich, Wagistrasse 10, 8952 Schlieren, Switzerland; ETH Zurich, Department of Biology, Wolfgang-Pauli-Strasse 27, 8093 Zurich, Switzerland; ETH Zurich, Department of Health Sciences and Technology, Otto-Stern-Weg 3, 8093 Zurich, Switzerland; University Hospital Basel and University of Basel, Department of Biomedicine, Hebelstrasse 20, 4031 Basel, Switzerland; University of Zurich, Department of Quantitative Biomedicine, Winterthurerstrasse 190, 8057 Zurich, Switzerland; F. Hoffmann-La Roche Ltd, Grenzacherstrasse 124, 4070 Basel, Switzerland; ETH Zurich, Department of Biosystems Science and Engineering, Mattenstrasse 26, 4058 Basel, Switzerland; ETH Zurich, Department of Biology, Wolfgang-Pauli-Strasse 27, 8093 Zurich, Switzerland; ETH Zurich, Department of Computer Science, Institute of Machine Learning, Universitätstrasse 6, 8092 Zurich, Switzerland; Swiss Institute of Bioinformatics, Zurich, Switzerland; University Hospital Zurich, Biomedical Informatics, Schmelzbergstrasse 26, 8006 Zurich, Switzerland; University Hospital Basel, Institute of Medical Genetics and Pathology, Schönbeinstrasse 40, 4031 Basel, Switzerland; ETH Zurich, Department of Biology, Wolfgang-Pauli-Strasse 27, 8093 Zurich, Switzerland; ETH Zurich, Department of Computer Science, Institute of Machine Learning, Universitätstrasse 6, 8092 Zurich, Switzerland; Swiss Institute of Bioinformatics, Zurich, Switzerland; University Hospital Zurich, Biomedical Informatics, Schmelzbergstrasse 26, 8006 Zurich, Switzerland; Swiss Institute of Bioinformatics, Zurich, Switzerland; ETH Zurich, Department of Biology, Wolfgang-Pauli-Strasse 27, 8093 Zurich, Switzerland; F. Hoffmann-La Roche Ltd, Grenzacherstrasse 124, 4070 Basel, Switzerland; ETH Zurich, Department of Biology, Wolfgang-Pauli-Strasse 27, 8093 Zurich, Switzerland; F. Hoffmann-La Roche Ltd, Grenzacherstrasse 124, 4070 Basel, Switzerland; University Hospital Basel, Spitalstrasse 21/Petersgraben 4, 4031 Basel, Switzerland; ETH Zurich, Department of Biosystems Science and Engineering, Mattenstrasse 26, 4058 Basel, Switzerland; University Hospital Basel, Department of Information- and Communication Technology, Spitalstrasse 26, 4031 Basel, Switzerland; University Hospital Basel, Brustzentrum, Spitalstrasse 21, 4031 Basel, Switzerland; ETH Zurich, Department of Biology, Wolfgang-Pauli-Strasse 27, 8093 Zurich, Switzerland; ETH Zurich, Department of Computer Science, Institute of Machine Learning, Universitätstrasse 6, 8092 Zurich, Switzerland; Swiss Institute of Bioinformatics, Zurich, Switzerland; University Hospital Zurich, Biomedical Informatics, Schmelzbergstrasse 26, 8006 Zurich, Switzerland; ETH Zurich, Department of Biology, Wolfgang-Pauli-Strasse 27, 8093 Zurich, Switzerland; ETH Zurich, Department of Health Sciences and Technology, Otto-Stern-Weg 3, 8093 Zurich, Switzerland; F. Hoffmann-La Roche Ltd, Grenzacherstrasse 124, 4070 Basel, Switzerland; Roche Diagnostics GmbH, Nonnenwald 2, 82377 Penzberg, Germany; University Hospital Zurich, Department of Medical Oncology and Hematology, Rämistrasse 100, 8091 Zurich, Switzerland; ETH Zurich, Department of Biology, Wolfgang-Pauli-Strasse 27, 8093 Zurich, Switzerland; ETH Zurich, Department of Computer Science, Institute of Machine Learning, Universitätstrasse 6, 8092 Zurich, Switzerland; Swiss Institute of Bioinformatics, Zurich, Switzerland; University Hospital Zurich, Biomedical Informatics, Schmelzbergstrasse 26, 8006 Zurich, Switzerland; ETH Zurich, Department of Biology, Wolfgang-Pauli-Strasse 27, 8093 Zurich, Switzerland; ETH Zurich, Department of Biosystems Science and Engineering, Mattenstrasse 26, 4058 Basel, Switzerland; University Hospital Zurich, Department of Gynecology, Frauenklinikstrasse 10, 8091 Zurich, Switzerland; Cantonal Hospital Baselland, Medical University Clinic, Rheinstrasse 26, 4410 Liestal, Switzerland; ETH Zurich, Department of Health Sciences and Technology, Otto-Stern-Weg 3, 8093 Zurich, Switzerland; ETH Zurich, Department of Biology, Wolfgang-Pauli-Strasse 27, 8093 Zurich, Switzerland; F. Hoffmann-La Roche Ltd, Grenzacherstrasse 124, 4070 Basel, Switzerland; University Hospital Basel, Institute of Medical Genetics and Pathology, Schönbeinstrasse 40, 4031 Basel, Switzerland; University of Zurich, Services and Support for Science IT, Winterthurerstrasse 190, 8057 Zurich, Switzerland; University Hospital Basel, Institute of Medical Genetics and Pathology, Schönbeinstrasse 40, 4031 Basel, Switzerland; University of Bern, Department of BioMedical Research, Murtenstrasse 35, 3008 Bern, Switzerland; University Hospital Basel, Institute of Medical Genetics and Pathology, Schönbeinstrasse 40, 4031 Basel, Switzerland; ETH Zurich, Department of Biology, Wolfgang-Pauli-Strasse 27, 8093 Zurich, Switzerland; F. Hoffmann-La Roche Ltd, Grenzacherstrasse 124, 4070 Basel, Switzerland; ETH Zurich, Department of Biology, Wolfgang-Pauli-Strasse 27, 8093 Zurich, Switzerland; University of Zurich, Institute of Molecular Life Sciences, Winterthurerstrasse 190, 8057 Zurich, Switzerland; University Hospital Basel and University of Basel, Department of Biomedicine, Hebelstrasse 20, 4031 Basel, Switzerland; University Hospital Basel, Institute of Medical Genetics and Pathology, Schönbeinstrasse 40, 4031 Basel, Switzerland; ETH Zurich, NEXUS Personalized Health Technologies, John-von-Neumann-Weg 9, 8093 Zurich, Switzerland; Swiss Institute of Bioinformatics, Zurich, Switzerland; University Hospital Basel and University of Basel, Department of Biomedicine, Hebelstrasse 20, 4031 Basel, Switzerland; Roche Pharmaceutical Research and Early Development, Roche Innovation Center Basel, Grenzacherstrasse 124, 4070 Basel, Switzerland; ETH Zurich, NEXUS Personalized Health Technologies, John-von-Neumann-Weg 9, 8093 Zurich, Switzerland; Swiss Institute of Bioinformatics, Zurich, Switzerland; ETH Zurich, Department of Biology, Wolfgang-Pauli-Strasse 27, 8093 Zurich, Switzerland; ETH Zurich, Department of Computer Science, Institute of Machine Learning, Universitätstrasse 6, 8092 Zurich, Switzerland; Swiss Institute of Bioinformatics, Zurich, Switzerland; University Hospital Zurich, Biomedical Informatics, Schmelzbergstrasse 26, 8006 Zurich, Switzerland; ETH Zurich, Department of Biosystems Science and Engineering, Mattenstrasse 26, 4058 Basel, Switzerland; University of Zurich, Institute of Molecular Life Sciences, Winterthurerstrasse 190, 8057 Zurich, Switzerland; Roche Pharmaceutical Research and Early Development, Roche Innovation Center Munich, Roche Diagnostics GmbH, Nonnenwald 2, 82377 Penzberg, Germany; Roche Pharmaceutical Research and Early Development, Roche Innovation Center Basel, Grenzacherstrasse 124, 4070 Basel, Switzerland; F. Hoffmann-La Roche Ltd, Grenzacherstrasse 124, 4070 Basel, Switzerland; ETH Zurich, Department of Biosystems Science and Engineering, Mattenstrasse 26, 4058 Basel, Switzerland; University Hospital Zurich, Clinical Trials Center, Rämistrasse 100, 8091 Zurich, Switzerland; ETH Zurich, NEXUS Personalized Health Technologies, John-von-Neumann-Weg 9, 8093 Zurich, Switzerland; Swiss Institute of Bioinformatics, Zurich, Switzerland; University of Zurich, Department of Quantitative Biomedicine, Winterthurerstrasse 190, 8057 Zurich, Switzerland; ETH Zurich, Department of Biology, Wolfgang-Pauli-Strasse 27, 8093 Zurich, Switzerland; ETH Zurich, Department of Biology, Wolfgang-Pauli-Strasse 27, 8093 Zurich, Switzerland; F. Hoffmann-La Roche Ltd, Grenzacherstrasse 124, 4070 Basel, Switzerland; ETH Zurich, NEXUS Personalized Health Technologies, John-von-Neumann-Weg 9, 8093 Zurich, Switzerland; Swiss Institute of Bioinformatics, Zurich, Switzerland; ETH Zurich, Department of Biology, Wolfgang-Pauli-Strasse 27, 8093 Zurich, Switzerland; ETH Zurich, Department of Computer Science, Institute of Machine Learning, Universitätstrasse 6, 8092 Zurich, Switzerland; Swiss Institute of Bioinformatics, Zurich, Switzerland; University Hospital Zurich, Biomedical Informatics, Schmelzbergstrasse 26, 8006 Zurich, Switzerland; ETH Zurich, NEXUS Personalized Health Technologies, John-von-Neumann-Weg 9, 8093 Zurich, Switzerland; Swiss Institute of Bioinformatics, Zurich, Switzerland; University Hospital Zurich, Department of Medical Oncology and Hematology, Rämistrasse 100, 8091 Zurich, Switzerland; ETH Zurich, Department of Biology, Wolfgang-Pauli-Strasse 27, 8093 Zurich, Switzerland; ETH Zurich, Department of Computer Science, Institute of Machine Learning, Universitätstrasse 6, 8092 Zurich, Switzerland; Swiss Institute of Bioinformatics, Zurich, Switzerland; University Hospital Zurich, Biomedical Informatics, Schmelzbergstrasse 26, 8006 Zurich, Switzerland; University Hospital Basel, Institute of Medical Genetics and Pathology, Schönbeinstrasse 40, 4031 Basel, Switzerland; Roche Pharmaceutical Research and Early Development, Roche Innovation Center Zurich, Wagistrasse 10, 8952 Schlieren, Switzerland; ETH Zurich, NEXUS Personalized Health Technologies, John-von-Neumann-Weg 9, 8093 Zurich, Switzerland; Swiss Institute of Bioinformatics, Zurich, Switzerland; ETH Zurich, Department of Biosystems Science and Engineering, Mattenstrasse 26, 4058 Basel, Switzerland; University Hospital Zurich, Department of Dermatology, Gloriastrasse 31, 8091 Zurich, Switzerland; ETH Zurich, Department of Health Sciences and Technology, Otto-Stern-Weg 3, 8093 Zurich, Switzerland; University Hospital Basel, Brustzentrum & Tumorzentrum, Petersgraben 4, 4031 Basel, Switzerland; University Hospital Basel, Institute of Medical Genetics and Pathology, Schönbeinstrasse 40, 4031 Basel, Switzerland; University Hospital Zurich, Clinical Trials Center, Rämistrasse 100, 8091 Zurich, Switzerland; University Hospital Basel and University of Basel, Department of Surgery, Brustzentrum, Spitalstrasse 21, 4031 Basel, Switzerland; ETH Zurich, Department of Biology, Wolfgang-Pauli-Strasse 27, 8093 Zurich, Switzerland; University Hospital and University of Zurich, Department of Neurology, Frauenklinikstrasse 26, 8091 Zurich, Switzerland; ETH Zurich, Department of Health Sciences and Technology, Otto-Stern-Weg 3, 8093 Zurich, Switzerland; ETH Zurich, Department of Biology, Wolfgang-Pauli-Strasse 27, 8093 Zurich, Switzerland; F. Hoffmann-La Roche Ltd, Grenzacherstrasse 124, 4070 Basel, Switzerland; Cantonal Hospital Baselland, Medical University Clinic, Rheinstrasse 26, 4410 Liestal, Switzerland; University Hospital Basel and University of Basel, Department of Biomedicine, Hebelstrasse 20, 4031 Basel, Switzerland; University Hospital Basel, Centre for Neuroendocrine & Endocrine Tumours, Spitalstrasse 21/Petersgraben 4, 4031 Basel, Switzerland; ETH Zurich, Department of Health Sciences and Technology, Otto-Stern-Weg 3, 8093 Zurich, Switzerland; ETH Zurich, NEXUS Personalized Health Technologies, John-von-Neumann-Weg 9, 8093 Zurich, Switzerland; Swiss Institute of Bioinformatics, Zurich, Switzerland; ETH Zurich, Department of Biology, Wolfgang-Pauli-Strasse 27, 8093 Zurich, Switzerland; ETH Zurich, Department of Health Sciences and Technology, Otto-Stern-Weg 3, 8093 Zurich, Switzerland; ETH Zurich, Department of Biology, Wolfgang-Pauli-Strasse 27, 8093 Zurich, Switzerland; ETH Zurich, Department of Computer Science, Institute of Machine Learning, Universitätstrasse 6, 8092 Zurich, Switzerland; Swiss Institute of Bioinformatics, Zurich, Switzerland; University Hospital Zurich, Biomedical Informatics, Schmelzbergstrasse 26, 8006 Zurich, Switzerland; ETH Zurich, Department of Biology, Wolfgang-Pauli-Strasse 27, 8093 Zurich, Switzerland; F. Hoffmann-La Roche Ltd, Grenzacherstrasse 124, 4070 Basel, Switzerland; University Hospital Zurich, Rämistrasse 100, 8091 Zurich, Switzerland; Department of Computer Science, ETH Zürich, 8092 Zürich, Switzerland; Swiss Institute of Bioinformatics, Quartier Sorge Bâtiment Amphipôle, 1015 Lausanne, Switzerland; Life Science Zurich Graduate School, PhD Program Molecular & Translational Biomedicine, 8057 Zürich, Switzerland; Department of Quantitative Biomedicine, University of Zürich, 8057 Zürich, Switzerland; Department of Biology, ETH Zürich, 8093 Zürich, Switzerland; Department of Computer Science, ETH Zürich, 8092 Zürich, Switzerland; Swiss Institute of Bioinformatics, Quartier Sorge Bâtiment Amphipôle, 1015 Lausanne, Switzerland; Life Science Zurich Graduate School, PhD Program Molecular & Translational Biomedicine, 8057 Zürich, Switzerland

## Abstract

**Motivation:**

Recent technological advances have led to an increase in the production and availability of single-cell data. The ability to integrate a set of multi-technology measurements would allow the identification of biologically or clinically meaningful observations through the unification of the perspectives afforded by each technology. In most cases, however, profiling technologies consume the used cells and thus pairwise correspondences between datasets are lost. Due to the sheer size single-cell datasets can acquire, scalable algorithms that are able to universally match single-cell measurements carried out in one cell to its corresponding sibling in another technology are needed.

**Results:**

We propose Single-Cell data Integration via Matching (SCIM), a scalable approach to recover such correspondences in two or more technologies. SCIM assumes that cells share a common (low-dimensional) underlying structure and that the underlying cell distribution is approximately constant across technologies. It constructs a technology-invariant latent space using an autoencoder framework with an adversarial objective. Multi-modal datasets are integrated by pairing cells across technologies using a bipartite matching scheme that operates on the low-dimensional latent representations. We evaluate SCIM on a simulated cellular branching process and show that the cell-to-cell matches derived by SCIM reflect the same pseudotime on the simulated dataset. Moreover, we apply our method to two real-world scenarios, a melanoma tumor sample and a human bone marrow sample, where we pair cells from a scRNA dataset to their sibling cells in a CyTOF dataset achieving 90% and 78% cell-matching accuracy for each one of the samples, respectively.

**Availability and implementation:**

https://github.com/ratschlab/scim.

**Supplementary information:**

Supplementary data are available at *Bioinformatics* online.

## 1 Introduction

The ability to dissect a tissue into its cellular components to study them individually or to investigate the interplay between the different cell-type fractions is an exciting new possibility in biological research that has already yielded important insights into the dynamics of various diseases including cancer (Chevrier *et al.*, 2017; Tirosh *et al.*, 2016). Recent advances in single-cell technologies enable molecular profiling of samples with greater granularity at the transcriptomic, proteomic, genomic as well as the functional assays level (Irmisch *et al.*, 2020; Rozenblatt-Rosen *et al.*, 2017). Each data modality produces different types and levels of information that need to be integrated and related to one another to truly grasp the mechanisms at play in the tissue microenvironment and to obtain a more comprehensive molecular understanding of the studied sample. Although technologies capable of measuring two modalities simultaneously are emerging (Stoeckius *et al.*, 2017; Zhu *et al.*, 2020), their scalability and widespread use are still limited. While multiple data integration tools have been developed recently, most approaches either depend on feature correspondences (Stuart *et al.*, 2019; Welch *et al.*, 2019) or are limited to a specific input type, for instance, scRNA and scDNA data (Campbell *et al.*, 2019; McCarthy *et al.*, 2020). To the best of our knowledge only two other approaches have been published (Amodio and Krishnaswamy, 2018; Welch *et al.*, 2017) with similar capabilities to Single-Cell data Integration via Matching (SCIM). MAGAN (Amodio and Krishnaswamy, 2018) is a Generative Adversarial Network capable of aligning the manifold between two technologies that relies on a feature correspondence loss. MATCHER (Welch *et al.*, 2017) is based on a Gaussian process latent variable model (GPLVM) (Lawrence, 2004) that can integrate technologies if their underlying latent structures can be represented in one dimension, applicable, for example, to model monotonic temporal processes. Other yet unpublished methods, such as MMD-MA (Liu *et al.*, 2019) and UnionCom (Cao *et al.*, 2020), rely on large kernel matrices which limit their scalability when using datasets of the sizes generally produced by molecular profiling.

**Fig. 1.**
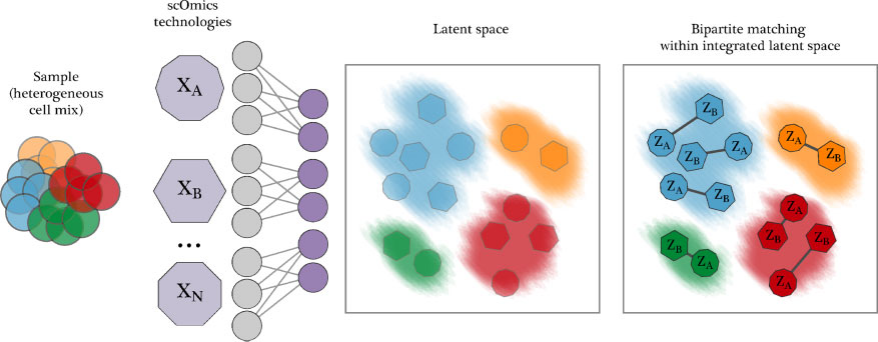
SCIM performs a pairwise matching of cell across multiple single-cell ’omics technologies. We assume that the input of each technology comes from the same (or similar) heterogeneous cell mix, depicted on the left. Technologies generate a set of single-cell ’omics datasets (violet polygons) in parallel (e.g. *X_A_*, *X_B_*, *X_N_*). These datasets are represented as matrices of cells-by-features, where features are specific to the profiling technology, but could be gene expression, protein levels, etc. SCIM proceeds to map cells into a technology-invariant latent space (left box) using an autoencoder framework and an adversarial term to keep technologies well integrated. Here, the latent representations capture the underlying structure in the cell mix (colored clouds) and analogous cells from different technologies (colored polygons) are placed in proximity. To integrate datasets, a fast bipartite matching scheme is applied, matching cells pairwise among datasets to cross-technology analogs, using their latent representations (right box)

Here, we propose SCIM, a method to match cells across different single-cell ’omics technologies. Our approach is universal, in the sense that it is in principle applicable to any single-cell technology and scales to arbitrary numbers of technologies. Further, we do not assume the existence of paired features between two technologies. This allows for the integration of technologies that measure for example the expression of a disjoint set of genes, or the integration of gene expression with image features as long as the underlying latent structure is present in those features. Our approach consists of two parts. First, we build an integrated latent space where representations are invariant to their corresponding technologies inspired by a model proposed previously (Yang and Uhler, 2019) and further extended in Yang *et al.* (2019). Then, we apply a cell-to-cell matching strategy that efficiently extracts cross-technology cell matches from the latent space. SCIM assumes a shared latent representation between technologies but, unlike other approaches, does not require one-to-one or overlapping correspondences between feature sets. Individual technologies often consume samples and thus, the input material provided to each profiling approach is typically an aliquot from a common sample cell suspension. Notwithstanding, given that the technology-specific datasets come from the same sample, (i.e. cell mix), expecting the same underlying distribution is an appropriate assumption. SCIM scales well in the number of cells in the input through the use of neural-nets, end-to-end training and an efficient bipartite matching algorithm. The training scheme allows for the addition of an arbitrary number of technologies, which can be trained in parallel (see Fig. 1).

## 2 Materials and methods

SCIM matches cells from a source technology to cells in one or multiple target technologies in two main steps. First, an integrated, technology-invariant latent space is produced using an encoder/decoder framework based on Yang and Uhler (2019). Then, cells are paired across different technologies via their latent representations using a version of the fast bipartite matching algorithm.

### 2.1 Model

Autoencoders produce low-dimensional representations of data by learning a pair of encoder and decoder functions, with parameters *ψ* and ϕ, respectively. The encoder maps input data into a lower-dimensional space, called the latent space, while the decoder tries to reconstruct the input data from its latent representation. The popular Variational Autoencoders (VAEs) take a generative approach to this problem (Kingma and Welling, 2013). Here, ϕ parameterizes the likelihood of the data given the latent representation $p_{\phi }(x|z)$ and *ψ* parameterizes the posterior probability of its latent representation $q_{\psi }(z|x)$. VAEs jointly learn ϕ and *ψ* to maximize a lower bound to the probability of the data p(x;ϕ,ψ), achieved in practice by minimizing

(1) $$L_{\mathrm{vae}}(\phi ,\psi ;x)=-\text{log}\;p_{\phi }(x|\hat{z})+D_{KL}(q_{\psi }(z|x)||p(z))$$

where $\hat{z}∼q_{\psi }(z|x),\;D_{KL}$ is the Kullback–Leibler (KL) divergence, and *p*(*z*) is a prior distribution over latent representations. Often *p*(*z*) and $q_{\psi }(z|x)$ are restricted to Gaussian forms since the KL divergence then has a closed-form solution.

#### 2.1.1 Constructing a technology-invariant latent space

SCIM encodes datasets into a shared latent space, which has ideally two properties. As in the VAE, inputs should be able to be reconstructed from their latent representations. In addition, the latent representations of each technology should be integrated well such that they are indistinguishable from each other. In a successful integration the resulting latent space will have corresponding cells across all technologies represented in close proximity.

To construct an integrated latent space, SCIM uses the following networks: a pair of encoder (*ψ_k_*) and decoder ($\phi_{k}$) networks for each technology *k* and a single discriminator network (*γ*) acting on the latent space. The discriminator is a binary classifier trained to identify the latent representation of a source technology from latent representations of all other technologies using a binary cross entropy loss.

SCIM yields an integrated latent space by minimizing the reconstruction error while adversarially fooling the discriminator. For notational brevity, we now let *ψ_k_* and $\phi_{k}$ also represent the probability distributions they parameterize. Given the measurements of a batch of cells from the target technology, *x_t_*, and the (fixed) latent representations of a batch of cells from the source technology, ${\hat{z}}_{s}∼\psi_{s}(x_{s})$, SCIM minimizes the following objective

(2) $$L(x_{t},{\hat{z}}_{s};\psi_{t},\phi_{t})=L_{\mathrm{nll}}(x_{t};\psi_{t},\phi_{t})+\beta L_{\mathrm{adv}}({\hat{z}}_{s},{\hat{z}}_{t};\psi_{t})$$

$L_{\mathrm{nll}}(x_{t};\phi_{t},\psi_{t})$ is the negative log-likelihood of the inputs under their reconstruction. $L_{\mathrm{adv}}$ is the discriminator’s classification error when trying to classify the latent representation samples ${\hat{z}}_{s}$/${\hat{z}}_{t}$ as the source/target technology. *β* is a hyperparameter weighing the influence of the adversarial loss. At the same time, *γ* is trained to correctly classify the technology of the ${\hat{z}}_{s}$ and ${\hat{z}}_{t}$ samples.

More intuitively, this framework can be seen as learning a VAE on each technology where the prior distribution is defined by the latent representations of the other technologies. $L_{\mathrm{adv}}$ can be interpreted as a divergence measure where, through the use of adversarial techniques, samples may be used in lieu of their potentially intractable probability distributions. Thus, the framework is equivalent to a set of Adversarial Autoencoders (Makhzani *et al.*, 2015) or Wasserstein Autoencoders (Tolstikhin *et al.*, 2017) which share a single discriminator.

#### 2.1.2 The orientation of latent space

Correctly orienting the latent space in an unsupervised manner is a challenging task (Locatello *et al.*, 2018; Yang and Uhler, 2019). Consider, for example, a simple monotonic temporal process. The latent representations for one dataset could be oriented from start to end, while another could be oriented from finish to start (Welch *et al.*, 2017). Equation 2 is satisfied, the representations are well integrated and inputs can be correctly reconstructed from them, yet the inter-dataset relationships are misaligned.

Makhzani *et al.* (2015) address a similar problem by concatenating one-hot representations of labels reflecting intra-technology structure (e.g. cell type is an appropriate choice for ’omics datasets) to the discriminator inputs, showing that this supervision is necessary to orient the latent space. Recently, Locatello *et al.* (2019) argued that only a small number of labels are actually needed to achieve orientation. To this end, we adopt a semi-supervised approach by adding a ‘censored’ label and randomly relabel cells in the training set.

#### 2.1.3 Model architecture

Unless specified otherwise, we adopt the following architecture settings. All networks use the ReLU activation. We set the latent dimension of all models to eight, but observed this choice to be flexible. We use discriminator networks with two layers and eight hidden units each. The Spectral Normalization framework (Miyato *et al.*, 2018) is used during training, which has been argued to stabilize discriminator training by effectively bounding its gradients. We use a Gaussian activation for all decoders, a 2 layer architecture with 64 hidden units for all simulated data networks, a 2 layer architecture with 8 hidden units for all CyTOF networks and a 2 layer architecture with 64 hidden units for all scRNA networks. The number of features and complexity of data is considered when choosing capacity and depth.

#### 2.1.4 Optimization

Optimization proceeds by iteratively fixing one technology as the source and one technology as the target. In the case of more than two technologies, the technology corresponding to the discriminator’s positive class must either be the source or target technology. The codes of the source technology are fixed and Equation 2 is minimized with gradient updates to the encoder and decoder, $\psi_{t}$ and *ϕ_t_*, of the target technology using gradients computed on the batch *x_t_*. After each update, the discriminator is trained to correctly classify ${\hat{z}}_{s}$ and ${\hat{z}}_{t}$. All networks in SCIM are optimized using the ADAM algorithm (Kingma and Ba, 2014).

We initialize SCIM by first training a VAE (Kingma and Welling, 2013) on a single source technology, and use the latent representations as the first set of ${\hat{z}}_{s}$. Unless specified otherwise the VAE is trained for 256 epochs using β=0.01 and a learning rate of 0.0005. A small value of *β* is needed for structure to be retained in the latent representations.

#### 2.1.5 Latent space evaluation and model selection

Due to the min–max nature of adversarial training, model comparison is challenging since one cannot directly compare the minimized objective functions of converged models (Lucic *et al.*, 2017). The computer vision community has introduced a number of metrics specific to the image domain to help compare models (Heusel *et al.*, 2017; Salimans *et al.*, 2016). Here, we need to validate the quality of a set of lower-dimensional latent representations.

Therefore, we use a k-Nearest Neighbor (kNN)-based divergence estimator (Wang *et al.*, 2009) to quantitatively evaluate the quality of the integrated latent space. The divergence score between two sets of codes *Z_s_* and *Z_t_* is calculated as:

(3) $$\hat{D}(Z_{s}||Z_{t})=\frac{1}{2}{\hat{D}}_{KL}(Z_{s}||Z_{t})+\frac{1}{2}{\hat{D}}_{KL}(Z_{t}||Z_{s})$$

where

(4) $${\hat{D}}_{KL}(P||Q)=\frac{d}{\left|P\right|}\sum_{p_{i}\in P}\;\text{log}\;\frac{\nu_{k}(p_{i})}{\rho_{k}(p_{i})}+\text{log}\;\frac{\left|P\right|}{|Q|-1}$$

where $\nu_{k}(p_{i})$ and $\rho_{k}(p_{i})$ are the distances from *p_i_* to the *k*th nearest neighbor in the sets *P* and *Q*, respectively and all $p_{i}\in R^{d}$. This estimator approximates a symmetric variant of a KL divergence, a measure of how much two distributions differ, using only empirical data. The divergence estimate is computed between the latent representations of the source technology and the target technology to measure the alignment of codes from the two technologies. Model selection can proceed at scale by selecting parameter configurations that align technology distributions and have low reconstruction error.

This approach draws inspiration from a proposed framework from Yang and Uhler (2019), where expression profiles are decoded from the latent space. We were able to utilize a kNN based divergence estimator (Wang *et al.*, 2019), to address typical problems in adversarial training. Further, SCIM does not decode values from latent space, but the low-dimensional representation is used solely to match the cells. Thus, the true observed marker abundances per cell pair, measured with different technologies, can be used for any downstream analysis. Moreover, the latent space matching may compensate for sub-optimal integration, providing an additional advantage over bare decoding.

### 2.2 Bipartite matching of latent representations

The obtained shared latent representation can be used for finding corresponding cells across technologies. Each cell is now characterized by a low-dimensional vector of latent codes, which are in one-to-one correspondence across technologies. First, the data is represented as a graph, where the nodes correspond to cells and edge weights correspond to the Euclidean distances between the cells in the latent space. To find the best pairwise matching efficiently, we phrase the task as a combinatorial bipartite matching problem (Ahuja *et al.*, 1993; Dell’Amico and Toth, 2000). In other words, the task is to identify edges connecting the cells that would result in a minimal total cost of all matches. In order to achieve this, we build a k-Nearest Neighbors (kNN) graph to identify a set of potential matches and reduce the complexity of the problem. Then, we extend the graph to account for single-cell data characteristics and solve the bipartite matching within a general framework of Minimum-Cost Maximum-Flow problems (Ahuja *et al.*, 1993; Klein, 1967).

#### 2.2.1 k-nearest neighbor approximation

Given the large number of cells in single-cell datasets, we reduce the search space to the *k* most likely potential matches. Two kNN graphs are built: (i) using source data queried by the target technology cells, and (ii) using target data queried by the source technology cells. A union of the established connections is used for further analysis. The sparsity of connections, regulated by the choice of hyperparameter *k*, corresponds to the trade-off between the computational performance (memory usage, run time) and the matching accuracy.

#### 2.2.2 Bipartite matching via Minimum-Cost Maximum-Flow

Based on a Euclidean cost matrix, we aim at finding the maximum number of cell pairs with minimum cost. This corresponds to finding a maximum flow that can be pushed through the graph, where each edge between cells has capacity 1, while minimizing the overall cost. To solve the Minimum-Cost Maximum-Flow problem in a computationally efficient way we use an implementation of the network simplex algorithm (Király and Kovács, 2012).

#### 2.2.3 Relaxation of one-to-one matching by graph extensions

**Fig. 2.**
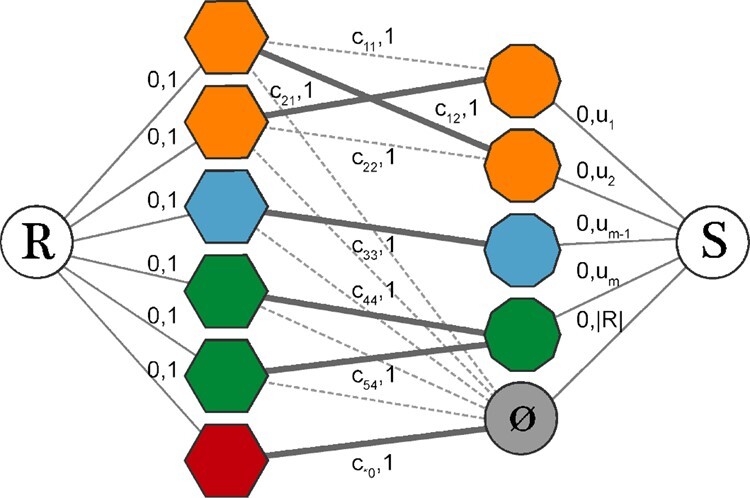
Fast bipartite matching using a customized Minimum-Cost Maximum-Flow framework. Nodes correspond to cells with technology represented by shape, i.e. hexagons and decagons. *R* and *S* represent root and sink nodes. Edges correspond to the sparse connections between the cells, resulting from a kNN search. Edge labels indicate matching cost (first value) and edge capacity (second value). Many-to-one matches in unbalanced datasets are enabled by increasing the capacities *u_i_* (for i∈1,…,m). The *null* node, colored in gray, captures matches of cells (from the bigger dataset on the left-hand side of the graph) that lack a close enough analog in the other technology. Its capacity equals the cardinality of the bigger dataset and the cost $c_{*0}$, i.e. null match penalty, is relatively high. The thicker lines linking the nodes represent the actual matches selected by the algorithm.

Bipartite matching approach makes the assumption that each cell has one and only one direct corresponding sibling in the other technology. To allow for mismatches due to expected variation in cellular composition, we expand the kNN graph with sparse connections by adding a densely connected *null* node with high capacity and high assignment cost. This allows to capture potentially poorly matched cells. The magnitude of the null match penalty corresponds to a given percentile *p* of the overall costs and is a hyperparameter. The extended graph structure is depicted in Figure 2, where *R* and *S* refer to the root and sink nodes, respectively. Furthermore, to account for differences in the number of cells between modalities (*n*, *m*), we allow for one-to-many matches by increasing the capacity of the edges incoming to the sink (*u_i_* for i∈{1,…,m}), assuming the nodes linked to the sink correspond to the smaller dataset (m≤n). To prevent all matches from collapsing onto a very small set of nodes, we constrain the incoming sink capacities, excluding the null node, to equal the cardinality of the bigger dataset divided by the cardinality of the smaller dataset, with the capacities distributed uniformly across the sink edges. If more than two data modalities are present, the bipartite matching is solved sequentially by obtaining pairwise matches between technologies.

#### 2.2.4 Matching evaluation

The quality of matching is evaluated on several levels. First, the accuracy corresponding to the fraction of true positives with regards to cell-type label is reported. Cell types can be determined in a technology-specific manner and the accuracy is reported on a common denominator. If more fine-grained cellular information is available, such as pseudotime, a direct comparison of this quantity is carried out. Furthermore, in real-world data settings we utilize the raw marker expression to investigate correspondence of the matched cells. Namely, Spearman’s and Pearson’s correlation coefficients are computed between the expression values across matches.

## 3 Data

### 3.1 Simulated data

**Fig. 3.**
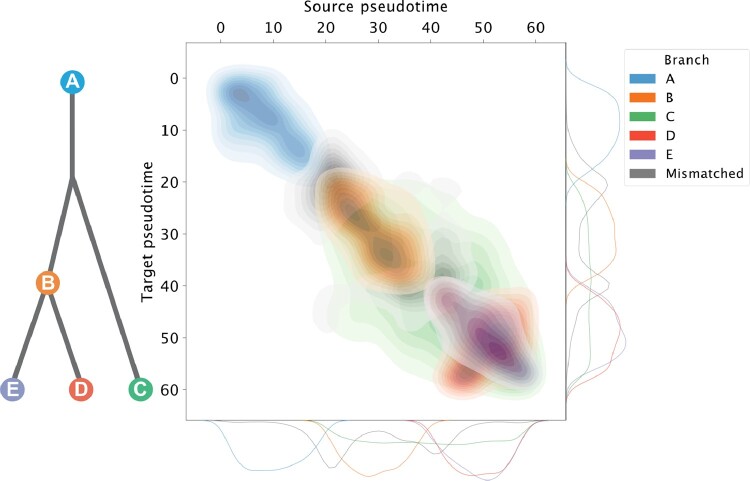
Evaluation of cross-technology cell matches made by SCIM on the simulated data. The tree defining the temporal branching process underlying the simulated data is shown on the left. Cells are matched across datasets pairwise using the bipartite matching scheme and the results are depicted on the right hand-side. The Results are shown as a density plot of pseudotime values across matched cells between the source technology (*x*-axis) and the target technology (*y*-axis). Cells matched to the same branch label are colored according to the branch-color scheme (accuracy: 86%), while mismatches are depicted in gray and appear mostly in the branching points. Marginal distributions of cell pseudotime for each branch are shown at the bottom (source technology) and left (target technology) of the density plot. We report a correlation of 0.83 (Spearman) and 0.86 (Pearson) for pseudotime label pairs

Using PROSSTT (Papadopoulos *et al.*, 2019), we generate three single-cell ’omics-styled technologies which share a common latent structure without direct feature correspondences. PROSSTT parameterizes a negative binomial distribution given a tree representing an underlying temporal branching process. By using the same tree and running PROSSTT under different seeds, we obtain three datasets with a common latent structure yet lacking any correspondences between features. We used a five branch tree with different branch lengths (Fig. 3). Each dataset contains 64 000 cells with 256 markers. The simulated datasets are available under http://tu-pro.ch/download/scim//.

### 3.2 Single-cell profile of a melanoma patient

The motivating dataset for our research questions is generated by the Tumor Profiler (TuPro) Consortium (Irmisch *et al.*, 2020) as part of a multi-center, multi-cancer study comprising metastatic tumors from a cohort of deeply phenotyped individuals. Each patient’s data is analyzed with multiple technologies, including scRNA-sequencing (Tang *et al.*, 2009) and Cytometry by Time Of Flight (Bandura *et al.*, 2009, CyTOF), all capable of dissecting the tumor microenvironment and providing single-cell level, complementary information about the sample of interest. Although cell identity is lost throughout the experimental process, the cells investigated by both technologies stem from the same population (i.e. were obtained from an aliquot of a common cell suspension). 

#### 3.2.1 CyTOF data preparation

The patient’s sample was profiled with CyTOF using a 41-markers panel designed for an in-depth characterization of the immune compartment of a sample. Data preprocessing was performed following the workflow described in Chevrier *et al.* (2017, 2018). Cell-type assignment was performed using a Random Forest classifier trained on multiple manually gated samples. To investigate the utility of SCIM, we considered a subset comprising B-Cells and T-Cells only, for a total of n=135 334 cells (see Table 1). This dataset is further referred to as *target* dataset.

#### 3.2.2 scRNA data preparation

A second aliquot of the same patient sample was analyzed by droplet-based scRNA-sequencing using the 10× Genomics platform. A detailed description of the data analysis workflow is beyond the scope of this work and will be published elsewhere. In brief, standard QC-measures and preprocessing steps, such as removal of low quality cells, as well as filtering out mitochondrial, ribosomal and non-coding genes, were applied. Expression data was library-size normalized and corrected for the cell-cycle effect. Cell-type identification was performed using a set of cell-type-specific marker genes (Tirosh *et al.*, 2016). Genes were then filtered to retain those that could code for proteins measured in CyTOF channels, the top 32 T-Cell/B-Cell marker genes, and the remaining most variable genes for a final set of 256. The total number of B-Cells and T-Cells (see Table 1) in this dataset amounts to m=4683. The scRNA dataset is used as *source* dataset throughout the manuscript.

### 3.3 Single-cell profile of human bone marrow

Oetjen *et al.* (2018) used several bulk and single-cell technologies to comprehensively characterize human bone marrow. The data was obtained from 20 healthy donors, whereas all data modalities were acquired for 8 samples. For our application we consider the single-cell transcriptome profile as well as CyTOF measurements of sample *O* from this dataset, that were carried out with the objective of describing in detail a T-Cell population. The data was preprocessed as described in Oetjen *et al.* (2018). The cell-type information for scRNA data was obtained directly by the courtesy of the authors, whereas CyTOF cells were manually gated using the strategy presented in Supplementary Figure S8. A subpopulation of CD8 naive T-Cells was filtered out due to a very small number of cells. The preprocessed data of the analyzed sample included several T-Cell subtypes (see Table 2).

## 4 Experiments

### 4.1 Three technology simulated data

We apply SCIM to integrate the three simulated datasets. The discriminator is trained to classify the source technology and is fully supervised using the branch label. The latent space is initialized by training a VAE on the source technology. The latent representations of the source technology are fixed, and the two target technologies are trained for 256 epochs. Bipartite matching is performed for each pair of datasets, using *k *=* *64 and a null match penalty set to the 95th percentile of edge costs.

### 4.2 Integration of scRNA and CyTOF patient data

We apply SCIM to integrate two sets of scRNA/CyTOF data, one set corresponds to a melanoma tumor from the Tumor Profiler project (Irmisch *et al.*, 2020) and the other one to a human bone marrow sample from Oetjen *et al.* (2018). The scRNA technology was chosen both times as the source technology, and the latent space is initialized by training a VAE. SCIM is trained for 64 epochs to integrate the CyTOF technologies. The discriminator is trained in both cases to classify the source technology. We used a semi-supervised strategy and use only 10% of the cell-type labels to help orienting the latent space. Bipartite matching is performed in both cases using *k *=* *50 and a null match penalty set to the 95th percentile of edge costs.

## 5 Results

We evaluate the SCIM framework on a simulated dataset based on PROSSTT (Papadopoulos *et al.*, 2019) as well as two real-world settings, where we match cells from CyTOF and scRNA measurements taken from a single sample analyzed within the Tumor Profiler project (Irmisch *et al.*, 2020) and from a human bone marrow sample (Oetjen *et al.*, 2018). We provide an implementation of the proposed approach in python using TensorFlow (Abadi *et al.*, 2015).

### 5.1 SCIM aligns substructure in simulated data

**Fig. 4.**
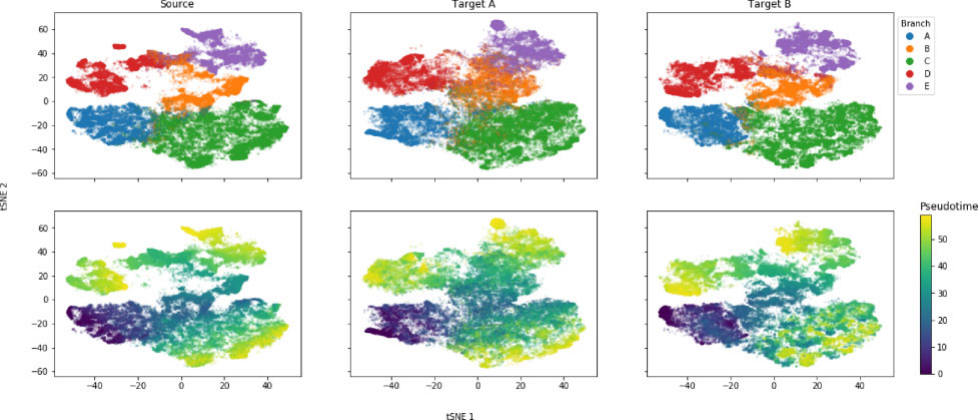
Integrated latent space of three synthetic datasets. Three single-cell ’omics datasets (Source, Target A and Target B) are generated (Papadopoulos *et al.*, 2019) from a shared underlying temporal branching process (as defined in Fig. 3). The same branching process was used in all three cases, but the parameters governing their feature distributions are drawn with different seeds. Hence, their latent structure is the same, yet they share no correspondences between features. SCIM is run, fully supervised using the branch label, and all datasets are embedded into a shared latent space. tSNE embeddings (Maaten and Hinton, 2008) are computed and visualized on the combined latent representations from all three datasets. Each column shows only the cells from a single technology. In the top row cells are colored by their branch label, as indicated on the legend. In the bottom row, the cells are colored by their pseudotime, as indicated on the color bar on the right-hand side

Branches in PROSSTT define an overarching structure that mimics cell-types, while the temporal component, i.e. pseudotime, provides a continuous interpolation from one branch to another as described by the tree (Fig. 3). In latent space, the branch structure within the data produces large clusters, while the pseudotime structure provides orientation within each cluster as well as a global smoothing of the manifold. SCIM is run to integrate the three simulated datasets producing a technology-invariant latent space (see Fig. 4). SCIM embeddings capture the branching process and furthermore correctly orient the substructure of most branches (see Fig. 3). We report 86% of matches retained the branch label and strong correlations among pseudotime (Pearson: 0.86, Spearman: 0.83) using a null node penalty of 95th percentile that controls the false/true positive trade-off (see Supplementary Fig. S3). Furthermore, most branch mismatches occur at the nodes of the tree, where the label is ambiguous due to the continuous nature of the temporal process (see Supplementary Table S3).

The SCIM framework can be applied to a many-technology setting, and we demonstrate this by obtaining pairwise matches between all three datasets. SCIM successfully aligns the cells, based on evaluations on pseudotime (see Supplementary Fig. S1) as well as branch label (see Supplementary Tables S4 and S5), even when using codes from such an extended latent space.

These results demonstrate that SCIM is capable of accurately identifying the best matching cells across multiple technologies, based on the shared latent representations in the presence of an underlying branching process but in the absence of paired features.

#### 5.1.1 MATCHER comparison: capturing complex latent structure

We compare SCIM to MATCHER (Welch *et al.*, 2017), which is, to the best of our knowledge, the only other published work that can integrate multi-modal ’omics datasets in the absence of direct feature correspondences. MATCHER, however, assumes a one-dimensional latent structure that cannot capture hierarchical relationships, such as the ones exhibited in the simulated PROSSTT data, and frequently found and studied in single-cell datasets. Moreover, MATCHER is built around a GPLVM (Lawrence, 2004), which limits its scalability. To this end, we set a budget of 48 h compute time and limit memory consumption to 40 Gb. Using the latent representations generated by MATCHER, we solve the bipartite matching problem setting the same hyperparameter configuration. MATCHER is unable to model the PROSSTT branching structure and is outperformed by SCIM with respect to matching (see Supplementary Tables S6 and S7 and Supplementary Fig. S2).

### 5.2 Universal divergence scales model selection in SCIM

To evaluate the performance of SCIM on real data and to gain a better understanding of the individual components of our framework, we apply SCIM on a melanoma tumor sample from the Tumor Profiler Consortium (Irmisch *et al.*, 2020). Model selection in the adversarial setting with real-world data is challenging since there is no metric that captures model performance, nor does one have access to any ground truth data to evaluate on. To help model selection, we use a universal divergence estimator (Wang *et al.*, 2009) to evaluate the quality of the integrated latent space (see Section 2, Supplementary Fig. S6). This score measures how well two sets of points are mixed, and it is computed pairwise between source and target technologies. An optimization is defined as successful if the divergence and reconstruction errors are below the empirically set thresholds. This allows the evaluation of many model settings at scale despite operating in the adversarial setting. We find that performance depends on tuning *β* and the learning rates for the discriminator and encoder/decoder networks (see Supplementary Table S2).

### 5.3 Modified bi-partite matching is scalable

Due to the large number of cells profiled with each individual technology per sample, we precede our bipartite matching with a kNN search (see Section 2.2). This reduces the problem complexity by *a priori* discarding redundant edges in the graph. Experiments on the real-world melanoma sample investigating the level of sparsity, governed by a hyperparameter *k*, show that using even a small number of neighbors provides good matching accuracy and performance saturates past *k *>* *100 (see Supplementary Fig. S4, Supplementary Table S1). This is in line with our expectations since a match to an extremely distant neighbor is hard to justify. In order to maintain a high degree of sparsity, without sacrificing matching accuracy, we use *k *=* *50 in all further experiments.

### 5.4 SCIM pairs cells across scRNA and CyTOF in a melanoma sample

**Fig. 5.**
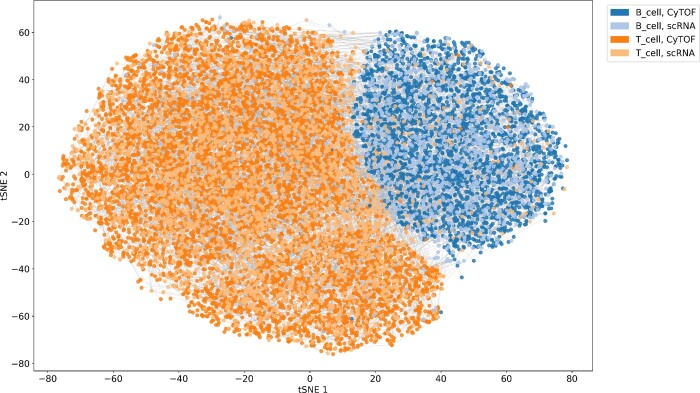
Integrated latent space and matches of scRNA and CyTOF cells from a melanoma sample from the Tumor Profiler Consortium. Discriminators are semi-supervised using 10% of the cell-type labels. Cells are colored by their cell-type label and shaded by their technology (dark shades: CyTOF, light shades: scRNA). Matches produced by SCIM are represented by gray lines connecting cells. tSNE embeddings (Maaten and Hinton, 2008) are computed on the whole dataset and then 10 000 matched pairs are sampled at random for visualization

Integrating data from scRNA and CyTOF technologies applied to a melanoma sample allows a multi-view perspective on cell dynamics and, thus, will eventually lead to a more thorough understanding of the underlying biological processes. Therefore we have evaluated the aforementioned melanoma sample with the SCIM framework. The bipartite matching on the latent codes has a 90% accuracy in recovering the cell-type label, calculated as the fraction of true positives over all matches. A more fine-grained visual evaluation is performed by inspecting the matches on a tSNE embedding of the integrated latent space marked by gray lines (see Fig. 5). Given different cell-type proportions in the data, a certain number of mismatches is expected, which corresponds to the lines joining points across the two cell types. The latent representation is explored thoroughly as 98%, and 99.9% of cells are matched to their analogs, from CyTOF and scRNA datasets, respectively. In comparison, a simple data-space matching approach would only utilize 29% of the scRNA cells (see Table 3 and Supplementary Fig. S5). To evaluate the latent space matching further, we used a more fine-grained information of marker expression correlation, to quantitatively assess the latent-space matching quality. We used the correlation coefficients between the expression of immunomarkers *CD20* and *CD3*. Both markers are characteristic for a subset of our data, as they are used to differentiate B-Cells and T-Cells, respectively. We found that matching using shared latent representations provides relatively high correlation coefficients (Pearson: 0.63 *CD20* and 0.51 for *CD3* marker, see Supplementary Fig. S7), given the expected low correlation between RNA expression and protein abundance. In conclusion, even in the presence of a subset of paired features across the technologies, using the shared latent representations proves beneficial for finding cell analogs.

**Table 1.** Characteristics of the preprocessed dataset derived from the melanoma sample

Dataset	No. of markers	No. of cells	T cells	B cells
CyTOF	41	135 334	70%	30%
scRNA	256	4683	73%	27%

**Table 2.** Characteristics of the preprocessed dataset derived from the human bone marrow sample

Dataset	No. of markers	No. of cells	CD8 effector	CD4 naive	CD4 memory
CyTOF	34	98 799	57%	26%	17%
scRNA	256	2388	46%	31%	23%

**Table 3.** The fraction of the cells from the source (scRNA) and target (CyTOF) datasets in TuPro that are matched using the Minimum-Cost Maximum-Flow algorithm

Space	No. of cells matched	Fraction	No. of cells matched	Fraction
	(source)	(source)	(target)	(target)
Latent	4680	0.999	133 130	0.98
Data	1342	0.29	28 178	0.21

*Note*: Only non-null matches are considered. The matching is performed using the shared latent codes or the corresponding features in the data space. The data-space matching results in all the matches collapsing onto very few cells (29% of the source dataset). Using latent codes allows for exploration of the whole space and providing best matches for almost all the cells.

### 5.5 SCIM recovers T-cell subpopulations across multi-modal human bone marrow data

We use SCIM to integrate T-Cells derived from one sample in the Human Bone Marrow study, profiled with scRNA and CyTOF technologies. The tSNE embedding based on the latent space codes implies good integration across technologies while preserving the cell-subtype structure Supplementary Figure S9. We evaluate the quality of matches using fine-grained labels indicating one of the T-Cell subtypes identified in gating: CD8 effector, CD4 naive, CD4 memory (see Section 3.3). In a fully supervised approach, using the labels to orient the latent space, we achieve an accuracy of 83% with less than 8% of matches directed to the null node. Nevertheless, when utilizing only 10% of the labels in the semi-supervised approach, we note only a slight drop in performance, obtaining 78% correct matches with less than 8% of cells directed to the null node. Evaluating on higher-level labels of CD8 versus CD4 T-Cells improves the accuracy to 91 and 86% for the fully supervised and semi-supervised approach, respectively. As expected, distinguishing cellular subtypes (e.g. CD4 naive versus CD4 memory) is more challenging due to high similarity between the cell populations, but overall SCIM is capable of accurately recovering even such subtle differences between cell types and states.

## 6 Discussion

We have developed SCIM, a new technology-invariant approach that pairs single-cell measurements across multi-modal datasets, without requiring feature correspondences. This development enables real multi-modal single-cell analysis, and opens up new opportunities to gain a multi-view understanding of the dynamics of individual cells in various disease or developmental states. The underlying autoencoder framework, combined with a customized bipartite matching approach, ensures scalability even to large numbers of cells.

We demonstrate that our model performs well on simulated data as well as on real-world melanoma and bone marrow samples profiled with scRNA and CyTOF. The SCIM framework is presented here on two and three data modalities and easily extends to additional technologies, providing a new and effective solution to the multi-level data integration problem. Integration of Image Mass Cytometry (Giesen *et al.*, 2014) or single-cell ATAC-seq data (Buenrostro *et al.*, 2015) for example, could enable the spatial analysis of regulatory and global expression changes not just in cancer but also in other diseases such as multiple sclerosis, where detailed spatiotemporal information has already been shown to provide relevant insights (Ramaglia *et al.*, 2019). Notably, SCIM allows for the integration of cell populations undergoing branching processes, enabling the study of temporal phenomena, such as developmental and cell fate determination. The scalability of our framework ensures applicability beyond single samples, facilitating the study of large cohorts or the integration of SCIM into analytical workflows.

As the low-dimensional representation of the data produced by SCIM is used solely to perform cell matching, it can be combined with any other analytical methods. The truly observed signals per cell pair measured with different technologies can be used for any downstream analysis. By adopting a divergence measure (Wang *et al.*, 2009), we addressed common constraints in adversarial training, such as training instability and convergence problems. To ensure scalability, we used a modified bipartite matching solution to efficiently match corresponding cells across technologies. Our extensions guarantee wider applicability of SCIM, since shifts in cell-type composition across disjoint aliquots, even coming from the same sample, can be expected. Furthermore, the introduction of the *null* node ensures a higher quality of matches by avoiding forced mismatches and thus, improving confidence in the cell-to-cell assignments. With increasing data dimensionality, the number of nearest neighbors (*k*) should also rise, since more ties are likely to occur. Nevertheless, the difference in the number of true positives across various values of *k* for the same dataset remains within 6% in our experiments. Hence, we can state that performance is robust against the choice of this hyperparameter. Depending on the actual data, bounded or unbounded edge capacities (nearest neighbor approach) may be preferable. For completeness, we provide the corresponding results with unbounded edge capacities in the Supplementary Material (Supplementary Tables S8–S15). Furthermore, SCIM is itself inherently modular, and other matching strategies that may be more suitable to other data types or experimental designs can be easily deployed on the integrated latent codes.

SCIM helps bridge the gap between data generation and integrative interpretation of diverse multi-modal data in the rapidly expanding field of single-cell biology, providing users with an easily scalable algorithm designed to maximize the information it provides and not limited to fit a particular analytical approach.

## Supplementary Material

btaa843_Supplementary_Material

## References

[btaa843-B1] Abadi M. et al (2015) TensorFlow: Large-scale machine learning on heterogeneous systems. Software available from tensorflow.org.

[btaa843-B2] Ahuja R.K. et al (1993). Network Flows: Theory, Algorithms, and Applications. Prentice-Hall, Inc., USA.

[btaa843-B3] Amodio M. and Krishnaswamy S. 2018. MAGAN: aligning biological manifolds. In Proceedings of the 35th International Conference on Machine Learning, PMLR, Vol. 80. pp. 215–223. July 10th-15th Stockholm,Sweden. http://proceedings.mlr.press/v80/amodio18a.html.

[btaa843-B4] Bandura D.R. et al (2009) Mass cytometry: technique for real time single cell multitarget immunoassay based on inductively coupled plasma time-of-flight mass spectrometry. Anal. Chem., 81, 6813–6822. 19601617 10.1021/ac901049w

[btaa843-B5] Buenrostro J.D. et al (2015) Single-cell chromatin accessibility reveals principles of regulatory variation. Nature, 523, 486–490. 26083756 10.1038/nature14590 PMC4685948

[btaa843-B6] Campbell K.R. et al (2019) clonealign: statistical integration of independent single-cell RNA and DNA sequencing data from human cancers. Genome Biol., 20, 54. 30866997 10.1186/s13059-019-1645-z PMC6417140

[btaa843-B7] Cao K. et al (2020) Unsupervised topological alignment for single-cell multi-omics integration. Bioinformatics. 36, i48–i56. 32657382 10.1093/bioinformatics/btaa443 PMC7355262

[btaa843-B8] Chevrier S. et al (2017) An immune atlas of clear cell renal cell carcinoma. Cell, 169, 736–749.e18. 28475899 10.1016/j.cell.2017.04.016 PMC5422211

[btaa843-B9] Chevrier S. et al (2018) Compensation of signal spillover in suspension and imaging mass cytometry. Cell Syst., 6, 612–620.e5. 29605184 10.1016/j.cels.2018.02.010 PMC5981006

[btaa843-B10] Dell'Amico M. , Toth P. (2000) Algorithms and codes for dense assignment problems: the state of the art. Discret. Appl. Math., 100, 17–48.

[btaa843-B11] Giesen C. et al (2014) Highly multiplexed imaging of tumor tissues with subcellular resolution by mass cytometry. Nat. Methods, 11, 417–422. 24584193 10.1038/nmeth.2869

[btaa843-B12] Heusel M. et al (2017) GANs trained by a two time-scale update rule converge to a local nash equilibrium. In: *Advances in Neural Information Processing Systems*, 30, pp. 6626–6637 December 4th - December 9th, Long Beach, CA, USA, [arXiv:1706.08500v6]

[btaa843-B13] Irmisch A. et al (2020) The tumor profiler study: integrated, multi-omic, functional tumor profiling for clinical decision support. medRxiv*.* doi:10.1101/2020.02.13.20017921. 10.1016/j.ccell.2021.01.004 33482122

[btaa843-B14] Kingma D.P. , Ba J. (2014) Adam: a method for stochastic optimization. arXiv.[arXiv:1412.6980]

[btaa843-B15] Kingma D.P. , Welling M. (2013) Auto-encoding variational Bayes. arXiv. [arXiv:1312.6114]

[btaa843-B16] Király Z. , Kovács P. (2012) Efficient implementations of minimum-cost flow algorithms. Acta Univ. Sapientiae Inf., 4, 67–118.

[btaa843-B17] Klein M. (1967) A primal method for minimal cost flows with applications to the assignment and transportation problems. Manag. Sci., 14, 205–220.

[btaa843-B18] Lawrence N.D. (2004) Gaussian process latent variable models for visualisation of high dimensional data. In: Thrun S. et al (eds.) Advances in Neural Information Processing Systems. Vol. 16. MIT Press, pp. 329–336, December 13th - December 18th, Vancouver, British Columbia, Canada.

[btaa843-B19] Liu J. et al (2019) Jointly embedding multiple single-cell omics measurements. bioRxiv, doi:10.1101/644310. 10.4230/LIPIcs.WABI.2019.10 PMC8496402 34632462

[btaa843-B20] Locatello F. et al (2018) Challenging common assumptions in the unsupervised learning of disentangled representations. In *Proceedings of the 36th International Conference on Machine Learning*, pp. 4114–4124, July 10th - July 15th, Long Beach, CA, USA. [arXiv:1811.12359].

[btaa843-B21] Locatello F. et al (2019) Disentangling factors of variation using few labels. In: Eights International Conference on Learning Representations. [arXiv: 1905.01258], May 6th - May 9th, New Orleans, Louisiana, USA

[btaa843-B22] Lucic M. et al (2017) Are GANs created equal? A large-scale study. In: *Advances in Neural Information Processing Systems*. pp. 700–709, Decmber 3rd - December 8th. Montreal, Canada. [arXiv:1711.10337].

[btaa843-B23] Maaten L.v.d. , Hinton G. (2008) Visualizing data using t-SNE. J. Mach. Learn. Res., 9, 2579–2605.

[btaa843-B24] Makhzani A. et al (2015) Adversarial autoencoders [arXiv:1511.05644].

[btaa843-B25] McCarthy D.J. et al; HipSci Consortium. (2020) Cardelino: computational integration of somatic clonal substructure and single-cell transcriptomes. Nat. Methods, 17, 414–421. 32203388 10.1038/s41592-020-0766-3

[btaa843-B26] Miyato O , et al (2018) Spectral normalization for generative adversarial networks. In: *Sixth International Conference on Learning Representations*, April 30–May 3 2018, Vancouver. [arXiv:1802.05957].

[btaa843-B27] Oetjen K.A. et al (2018) Human bone marrow assessment by single-cell RNA sequencing, mass cytometry, and flow cytometry. JCI Insight, 3, e124928. 30518681 10.1172/jci.insight.124928 PMC6328018

[btaa843-B28] Papadopoulos N. et al (2019) PROSSTT: probabilistic simulation of single-cell RNA-seq data for complex differentiation processes. Bioinformatics, 35, 3517–3519. 30715210 10.1093/bioinformatics/btz078 PMC6748774

[btaa843-B29] Ramaglia V. et al (2019) Multiplexed imaging of immune cells in staged multiple sclerosis lesions by mass cytometry. eLife, 8, e48051. 31368890 10.7554/eLife.48051 PMC6707785

[btaa843-B30] Rozenblatt-Rosen O. et al (2017) The human cell atlas: from vision to reality. Nat. News, 550, 451–453. 10.1038/550451a 29072289

[btaa843-B31] Salimans T. et al (2016) Improved techniques for training GANs. In: Lee D.D. et al (eds.) Advances in Neural Information Processing Systems. Vol. 29. Curran Associates, Inc., pp. 2234–2242, December 5th - December 10th, Barcelona, Spain.

[btaa843-B32] Stoeckius M. et al (2017) Simultaneous epitope and transcriptome measurement in single cells. Nat. Methods, 14, 865–868. 28759029 10.1038/nmeth.4380 PMC5669064

[btaa843-B33] Stuart T. et al (2019) Comprehensive integration of single-cell data. Cell, 177, 1888–1902.e21. 31178118 10.1016/j.cell.2019.05.031 PMC6687398

[btaa843-B34] Tang F. et al (2009) mRNA-Seq whole-transcriptome analysis of a single cell. Nat. Methods, 6, 377–382. 19349980 10.1038/nmeth.1315

[btaa843-B35] Tirosh I. et al (2016) Dissecting the multicellular ecosystem of metastatic melanoma by single-cell RNA-seq. Science, 352, 189–196. 27124452 10.1126/science.aad0501 PMC4944528

[btaa843-B36] Tolstikhin I. et al (2017) Wasserstein auto-encoders. In: Sixth International Conference on Learning Representations, April 30–May 3 2018, Vancouver. [arXiv:1711.01558].

[btaa843-B37] Wang Q. et al (2009) Divergence estimation for multidimensional densities via *k*-nearest-neighbor distances. IEEE Trans. Inf. Theory, 55, 2392–2405.

[btaa843-B38] Wang T. et al (2019) Bermuda: a novel deep transfer learning method for single-cell RNA sequencing batch correction reveals hidden high-resolution cellular subtypes. Genome Biol., 20, 165. 31405383 10.1186/s13059-019-1764-6 PMC6691531

[btaa843-B39] Welch J.D. et al (2017) Matcher: manifold alignment reveals correspondence between single cell transcriptome and epigenome dynamics. Genome Biol., 18, 138. 28738873 10.1186/s13059-017-1269-0 PMC5525279

[btaa843-B40] Welch J.D. et al (2019) Single-cell multi-omic integration compares and contrasts features of brain cell identity. Cell, 177, 1873–1887.e17. 31178122 10.1016/j.cell.2019.05.006 PMC6716797

[btaa843-B41] Yang, K. D. , Uhler C. (2019) Multi-domain translation by learning uncoupled autoencoders. In: *Computational Biology Workshop, International Conference on Machine Learning*. June 9–June 15 2019, Long Beach. [arXiv:1902.03515]

[btaa843-B42] Yang K.D. et al (2019) Multi-domain translation between single-cell imaging and sequencing data using autoencoders. *bioRxiv. [https://doi.org/10.1101/2019.12.13.875922]* 10.1038/s41467-020-20249-2 PMC7782789 33397893

[btaa843-B43] Zhu C. et al (2020) Single-cell multimodal omics: the power of many. Nat. Methods, 17, 11–14. 31907462 10.1038/s41592-019-0691-5

