# Supplementary material for: SCIM: universal single-cell matching with unpaired feature sets
Source: Bioinformatics. 2020 Dec 29;36(Suppl 2):i919–27. doi: 10.1093/bioinformatics/btaa843 (PMC7773480; doi:10.1093/bioinformatics/btaa843)
Supplement: btaa843_Supplementary_Material [file btaa843_supplementary_material.pdf]

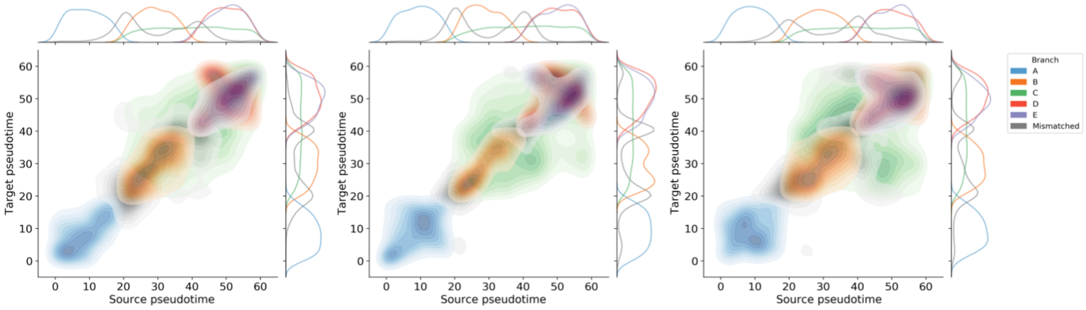

Fig. S1. Evaluation of cross-technology cell matches made by SCIM on the simulated data with three technologies. The pairwise matching is attained for Source-Target A, Source-Target B, and Target A-Target B, respectively. Here, we show a density plot for matched pseudotime values between the source technology and the target technology, colored by the branch label. Mismatched cells are colored in grey. The tree defining the temporal branching process can be found in Figure 3, left. Marginal distributions of cell pseudotime for each branch is shown on the top and right.

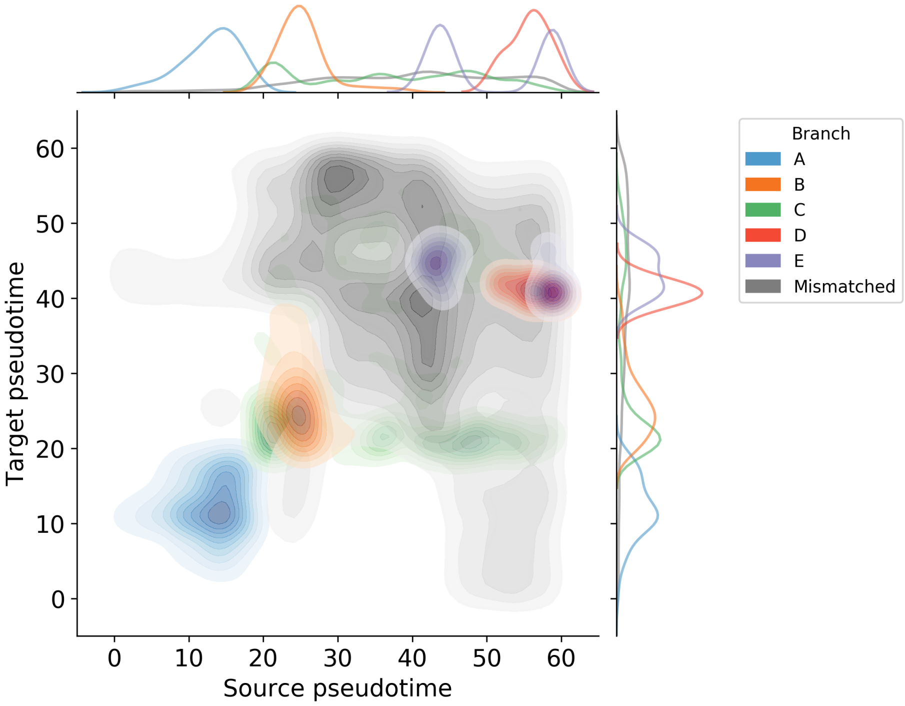

Fig. S2. Evaluation of cross-technology cell matches using latent representation obtained by MATCHER on the simulated data. Cells are matched across datasets pairwise using the bipartite matching scheme. Here we show a density plot for matched pseudotime values between the source technology and the target technology, colored by the branch label. Mismatched cells are colored in grey. Marginal distributions of cell pseudotime for each branch are shown on the top and right.

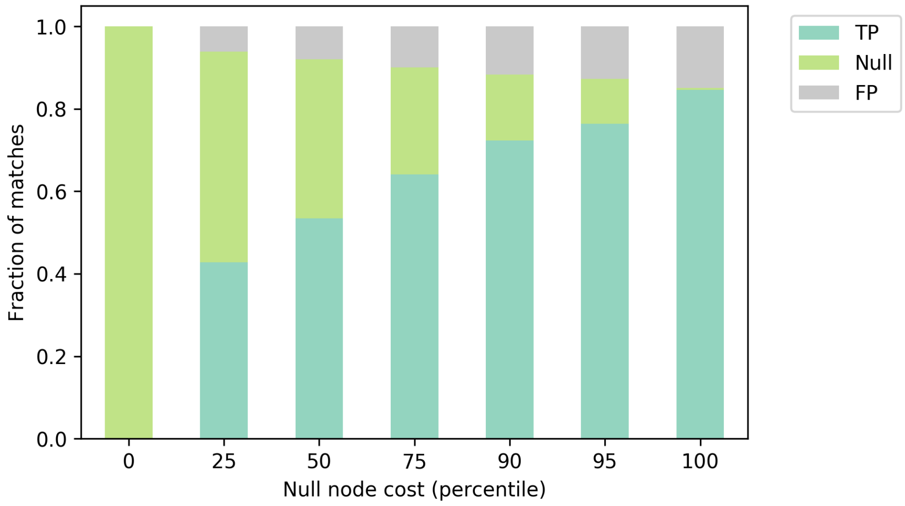

Fig. S3. True positive (TP), false positive (FP), and null matches fraction of all the matched pairs in the PROSSTT Source-Target experiment as a function of the cost for matching to the null node. The cost is specified as the percentile of the costs on all the other edges in the graph, i.e., Euclidean distance between latent codes of cells from source and target technologies. With decreasing null node cost, more false positives are removed, on the trade-off of removing more True Positives.

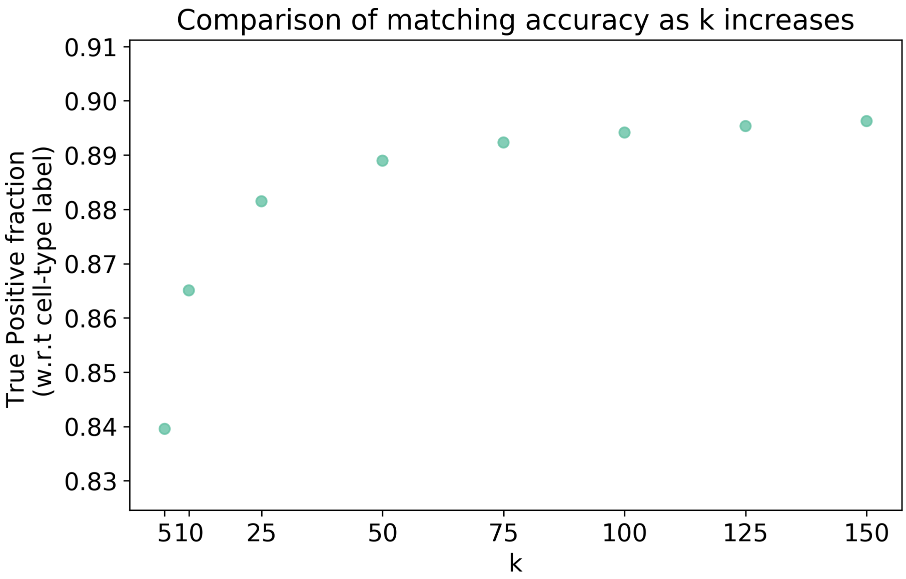

Fig. S4. Comparison of matching accuracy, with respect to cell-type label as less sparsity of connections, is imposed on the Tumor Profiler data. The number of considered Nearest Neighbors k is indicated on the x-axis, and the fraction of true positive matches, with respect to cell-type label, is depicted on the y-axis. The accuracy level saturates with $k = 100$.

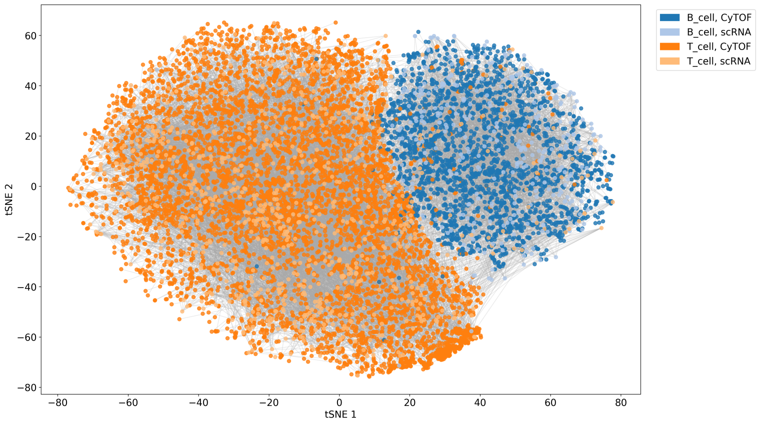

Fig. S5. Matches of scRNA and CyTOF cells from a melanoma patient from the Tumor Profiler Consortium computed on raw expression values. The latent space is constructed as in Figure 5, but matches are made using distances computed between the expression of 37 genes and proteins that could share a correspondence between datasets. 10,000 matched pairs are sampled at random for visualization.

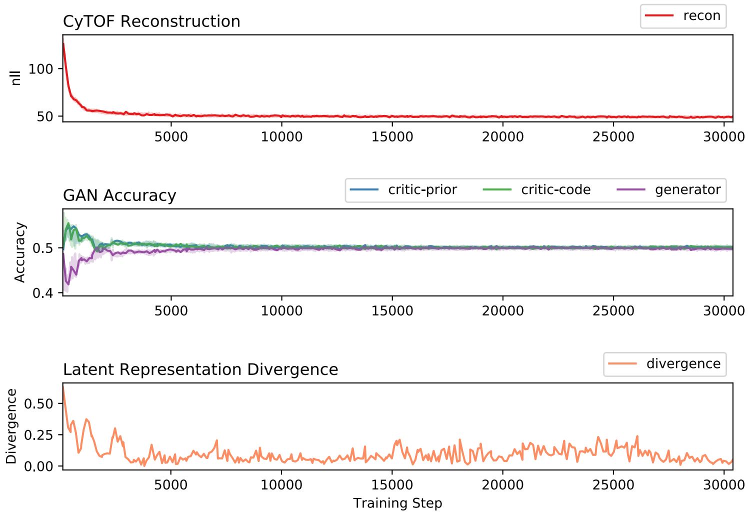

Fig. S6. Training progress of SCIM on a melanoma sample. The latent space is initialized by training a VAE on scRNA data. SCIM integrates CyTOF representations into the latent space defined by the scRNA codes. The top panel shows the negative log-likelihood of the CyTOF reconstruction. The middle panel shows the performance of the discriminator to correctly classify scRNA codes (critic-prior), CyTOF codes (critic-code), and the ability of the encoder to fool the discriminator, i.e., the misclassification accuracy (generator). The bottom panel shows the divergence of the latent representations. The model is able to converge quickly. However, the divergence score can fluctuate despite still fooling the discriminator. Training took 7815 seconds (just over 2 hours) and had a peak memory consumption of 1068 MB.

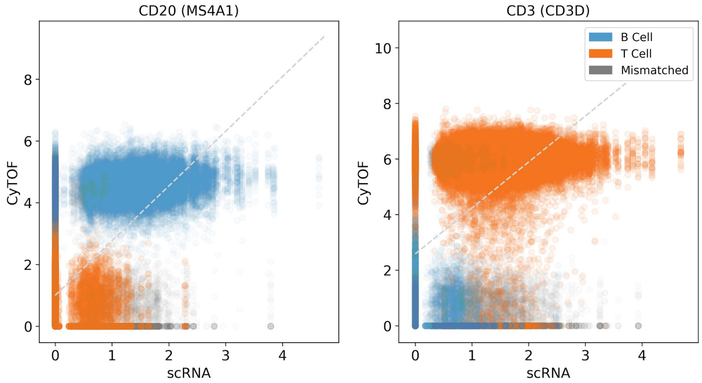

Fig. S7. CD20 and CD3 marker abundances measured with scRNA (gene, x-axis) and CyTOF (protein, y-axis) in a melanoma sample from the Tumor Profiler Consortium. The values on the axes represent normalized expression. Colors (blue, orange) represent cell types (B-Cell, T-Cell) while grey marks mismatches with respect to the cell-type label. The linear regression line is depicted by a dashed light grey line. Pearson’s correlation coefficient equals 0.63 and 0.51 for CD20 and CD3, respectively. Spearman’s correlation coefficient amounts to 0.55 and 0.42, for CD20 and CD3, respectively.

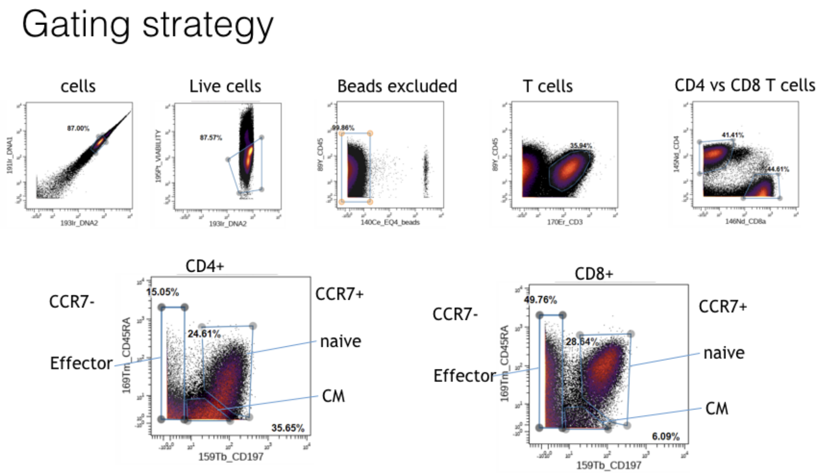

Fig. S8. Gating strategy for cells profiled with CyTOF from the bone marrow patient.

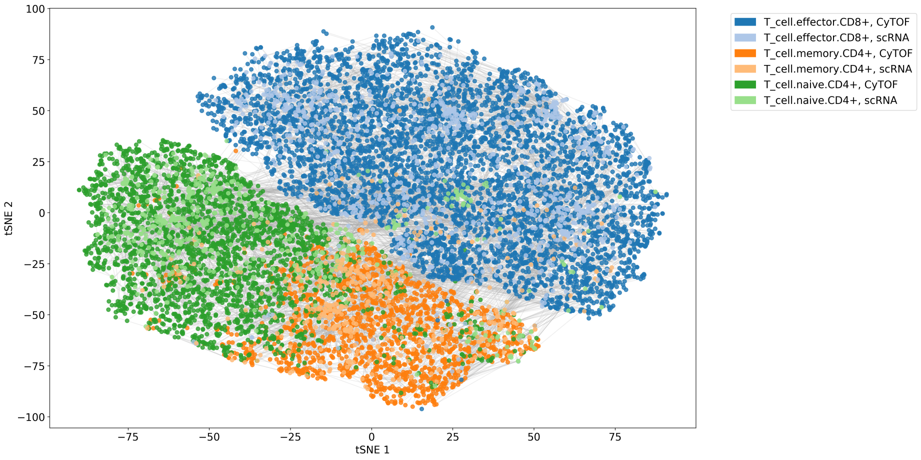

Fig. S9. A tSNE embedding (perplexity=30) of the integrated latent space with cell matches indicated by the grey lines. The shared representation was obtained in a semi-supervised fashion, utilizing 10% of the cell-type labels to orient the latent space. 10,000 matched pairs were sampled at random for the plot. Colors (blue, green, orange) represent T-Cell subtypes (CD8 effector, CD4 naive, CD4 memory), and color shades correspond to the profiling technology (light: scRNA, dark: CyTOF).

k	CPU time [s]	Max Memory [Mb]
5	1,492.67	1,674.94
10	2,803.33	2,456.27
25	7,723.67	4,690.05
50	7,441.33	8,740.34
75	12,656.00	12,344.52
100	17,331.00	17,002.75
125	26,047.00	20,612.78
150	33,718.00	24,222.75

Table S1. Memory usage and computation time of the bipartite matching as k hyperparameter in kNN search increases. The values were obtained on the whole TuPro dataset using the MCMF algorithm on an extended graph, as described in section **??**. The cost of matching to the null node was set to 95th percentile, and a union of connections obtained from kNN graphs with k indicated in the first column was used for matching. The memory and time reports were averaged across three independent runs.

Fraction Censored	β	Encoder/Decoders	Critic	Success Rate	Total Runs
0.00	16	0.0010	0.0010	14%	7
	16	0.0010	0.0005	14%	7
	16	0.0005	0.0005	10%	10
	16	0.0005	0.0010	10%	10
0.25	16	0.0010	0.0010	14%	14
	16	0.0005	0.0010	12%	17
	64	0.0001	0.0005	0%	7
	64	0.0001	0.0010	0%	7
0.75	16	0.0005	0.0010	17%	6
	32	0.0005	0.0005	12%	8
	16	0.0010	0.0005	8%	13
	64	0.0001	0.0005	0%	7
0.90	16	0.0005	0.0010	40%	10
	16	0.0010	0.0005	10%	10
	16	0.0005	0.0005	8%	12
	32	0.0010	0.0001	0%	10
0.95	32	0.0010	0.0001	0%	4
	64	0.0001	0.0005	0%	4
	64	0.0001	0.0010	0%	6
	32	0.0010	0.0010	0%	5
0.99	16	0.0005	0.0010	17%	6
	32	0.0010	0.0005	0%	6
	64	0.0001	0.0005	0%	4
	32	0.0010	0.0001	0%	6

Table S2. Ablation study for training SCIM on the melanoma patient at several levels of semi-supervision. Fraction censored is the fraction of labels removed during training. The top 4 configurations for each level of semi-supervision is shown. β is the regularization strength of the adversarial loss. Learning rates are the initial settings of the ADAM optimizer. If the latent space divergence is below 0.3 and the negative log-likelihood of the input under the reconstruction is below 47, the training is determined to be a success. These values were chosen empirically. We see that β and optimizer learning rates were heavily influential on model success.

Target A / Source	A	B	C	D	E
A	8,528	583	679	99	23
B	724	6,285	758	466	928
C	770	777	18,917	48	99
D	42	371	22	7,804	266
E	28	1,086	41	305	7,363

Table S3. Confusion table showing branch labels of the optimal matches of cells in the simulated PROSSTT data, between Source and Target A. Entries on the diagonal correspond to correct matches whereas off-diagonal elements to mismatches. The overall accuracy, with respect to the branch label, equals 86%.

Target B<br>Source	A	B	C	D	E
A	8,833	430	709	1	0
B	249	6,710	536	526	681
C	925	552	19,275	5	0
D	0	326	2	7,906	314
E	0	382	0	742	7,523

Table S4. Confusion table showing branch labels of the optimal matches of cells in the simulated PROSSTT data, between Source and Target B. Entries on the diagonal correspond to correct matches whereas off-diagonal elements to mismatches. The overall accuracy, with respect to the branch label, equals 89%.

Target B<br>Target A	A	B	C	D	E
A	7,718	738	844	21	19
B	518	6,312	785	679	1190
C	777	865	18,460	20	42
D	83	469	36	8,054	248
E	25	904	66	517	7,232

Table S5. Confusion table showing branch labels of the optimal matches of cells in the simulated PROSSTT data, between Target A and Target B. Entries on the diagonal correspond to correct matches whereas off-diagonal elements to mismatches. The overall accuracy, with respect to the branch label, equals 84%.

latent method	accuracy	#TP	#FP	#null matches	Spearman	Pearson
SCIM	86%	53,901	8,509	1,590	0.83	0.86
MATCHER	4%	1,443	37,047	25,510	-0.21	-0.19

Table S6. The matching results on the PROSSTT dataset, where the SCIM matching algorithm was applied to SCIM shared latent codes and the MATCHER latent representation. The table depicts accuracy with respect to branch label, the number of true (TP) and false positives (FP), as well as null node matches and the correlation coefficients for the pseudotime between matched source and target cells.

Target<br>Source	A	B	C	D	E
A	540	523	116	1,133	453
B	713	788	5,940	581	208
C	155	4,998	100	4,026	6,526
D	173	274	5,278	4	98
E	3,162	203	2,484	3	11

Table S7. Confusion table showing branch labels of the matched cells in the simulated PROSSTT data. SCIM matching algorithm was applied to a shared latent representation obtained with MATCHER. Entries on the diagonal correspond to correct matches whereas off-diagonal elements to mismatches. The overall accuracy, with respect to the branch label, equals 4%.

space	#cells matched (source)	fraction (source)	#cells matched (target)	fraction (target)
latent	4,652	0.993	134,930	0.997
data	236	0.05	130,899	0.967

Table S8. The fraction of the cells from the source (scRNA) and target (CyTOF) datasets in TuPro that are matched using the Minimum-Cost Maximum-Flow algorithm. Only non-null matches are considered. The matching is performed using the shared latent codes or the corresponding features in the data space. The bipartite matching was done with unbounded capacities on the sink edges. The data-space matching results in all the matches collapsing onto very few cells (5% of the source dataset). Using latent codes allows for exploration of the whole space and providing best matches for almost all the cells.

Target<br>Source	T_cell.effector.CD8+	T_cell.memory.CD4+	T_cell.naive.CD4+
T_cell.effector.CD8+	49,543	1,301	875
T_cell.memory.CD4+	3,351	13,483	5,439
T_cell.naive.CD4+	2,882	1,723	19,610

Table S9. Confusion table showing cell-type labels of the optimal matches of cells in the Human Bone Marrow data, between scRNA and CyTOF. The latent codes for matching were obtained in a semi-supervised approach, utilizing only 10% of cell-type labels to orient the latent space. The bipartite matching was done with unbounded capacities on the sink edges. Entries on the diagonal correspond to correct matches whereas off-diagonal elements to mismatches. The overall accuracy with respect to the cell-subtype label equals 84% and with respect to cell-type label equals 91%. The fraction of matched cells equals 0.82 and 0.99 for scRNA and CyTOF, respectively.

Target<br>Source	T_cell.effector.CD8+	T_cell.memory.CD4+	T_cell.naive.CD4+
T_cell.effector.CD8+	52,018	755	87
T_cell.memory.CD4+	3,438	10,901	2,689
T_cell.naive.CD4+	83	4,856	23,226

Table S10. Confusion table showing cell-type labels of the cell matches in the Human Bone Marrow data, between scRNA and CyTOF. The latent codes for matching were obtained in fully supervised approach, utilizing cell-type labels to orient the latent space. The bipartite matching was done with unbounded capacities on the sink edges. Entries on the diagonal correspond to correct matches whereas off-diagonal elements to mismatches. The overall accuracy with respect to the cell-subtype label equals 88% and with respect to cell-type label equals 96%. The fraction of matched cells equals 0.83 and 0.99 for scRNA and CyTOF, respectively.

Target A<br>Source	A	B	C	D	E
A	8,582	370	374	100	17
B	857	7,885	768	619	1,113
C	1,007	1,023	2,0064	115	169
D	29	273	17	9,140	205
E	26	1,040	74	334	8,388

Table S11. Confusion table showing branch labels of the optimal matches of cells in the simulated PROSSTT data, between Source and Target A, when using unbounded capacities on sink edges. Entries on the diagonal correspond to correct matches whereas off-diagonal elements to mismatches. The overall accuracy, with respect to the branch label, equals 86%. The fraction of matched cells equals 0.36 and 0.98 for Source and Target A technology reducing the number of matched cells substantially over the approach with restricted capacities.

Target B<br>Source	A	B	C	D	E
A	9,593	385	656	2	0
B	351	9,023	597	730	646
C	898	427	19,997	2	0
D	0	274	0	9,078	264
E	0	325	0	721	9,555

Table S12. Confusion table showing branch labels of the optimal matches of cells in the simulated PROSSTT data, between Source and Target B, when using unbounded capacities on sink edges. Entries on the diagonal correspond to correct matches whereas off-diagonal elements to mismatches. The overall accuracy, with respect to the branch label, equals 90%. The fraction of matched cells equals 0.32 and 0.99 for Source and Target A technology reducing the number of matched cells substantially over the approach with restricted capacities.

Target B<br>Target A	A	B	C	D	E
A	10,188	941	1,524	19	13
B	370	7,634	880	707	1,330
C	216	653	18,860	35	13
D	7	410	8	9,266	213
E	25	907	33	510	9,047

Table S13. Confusion table showing branch labels of the optimal matches of cells in the simulated PROSSTT data, between Target A and Target B, when using unbounded capacities on sink edges. Entries on the diagonal correspond to correct matches whereas off-diagonal elements to mismatches. The overall accuracy, with respect to the branch label, equals 86%. The fraction of matched cells equals 0.28 and 0.997 for Target A and Target B technology reducing the number of matched cells substantially over the approach with restricted capacities.

latent method	accuracy	#TP	#FP	#null matches	Spearman	Pearson
SCIM	86%	54,059	8,530	1,411	0.85	0.88
MATCHER	6%	3,858	60,096	46	-0.26	-0.28

Table S14. The matching results on the PROSSTT dataset, where the SCIM matching algorithm was applied to SCIM shared latent codes and the MATCHER latent representation. In both cases the capacities on sink edges were unbounded. The table depicts accuracy with respect to branch label, the number of true (TP) and false positives (FP), as well as null node matches and the correlation coefficients for the pseudotime between matched source and target cells.

Target<br>Source	A	B	C	D	E
A	811	770	236	1,725	2,142
B	2,197	2,551	9,448	1,315	479
C	243	6,518	481	7,616	7,914
D	688	650	8,116	4	175
E	6,604	212	3,047	1	11

Table S15. Confusion table showing branch labels of the matched cells in the simulated PROSSTT data. SCIM matching algorithm was applied to a shared latent representation obtained with MATCHER. In both cases the capacities on sink edges were unbounded. Entries on the diagonal correspond to correct matches whereas off-diagonal elements to mismatches. The overall accuracy, with respect to the branch label, equals 6%.

TUPRO Consortium

Rudolf Aebersold (2), Faisal S Al-Quaddoomi (8,15), Jonas Albinus (7), Ilaria Alborelli (23), Per-Olof Attinger (10), Marina Bacac (14), Daniel Baumhoer (23), Beatrice Beck-Schimmer (29), Niko Beerenwinkel (4), Christian Beisel (4), Lara Bernasconi (26), Anne Bertolini (8,15), Bernd Bodenmiller (33), Ximena Bonilla (3,6,15,25), Ruben Casanova (33), Stéphane Chevrier (33), Natalia Chicherova (8,15), Maya D'Costa (9), Esther Danenberg (35), Natalie Davidson (3,6,15,25), Reinhard Dummer (27), Stefanie Engler (33), Martin Erkens (12), Katja Eschbach (4), Cinzia Esposito (35), André Fedier (16), Pedro Ferreira (4), Joanna Ficek (3,6,15,25), Anja L Frei (28), Bruno Frey (11), Sandra Goetze (7), Linda Grob (8,15), Detlef Günther (5), Pirmin Haeuptle (1), Viola Heinzelmann-Schwarz (16,22), Sylvia Herter (14), Rene Holtackers (35), Tamara Huesser (14), Anja Irmisch (27), Francis Jacob (16), Andrea Jacobs (33), Tim M Jaeger (10), Katharina Jahn (4), Alva R James (3,6,15,25), Philip M Jermann (23), André Kahles (3,6,15,25), Abdullah Kahraman (15,28), Viktor H Koelzer (28), Werner Kuebler (24), Jack Kuipers (4), Christian P Kunze (21), Christian Kurzeder (19), Jelena Kühn-Georgijevic (12), Kjong-Van Lehmann (3,6,15,25), Mitchell Levesque (27), Sebastian Lugert (9), Gerd Maass (11), Sergio Maffioletti (34), Julien Mena (2), Ulrike Menzel (4), Nicola Miglino (1), Emanuela S Milani (7), Holger Moch (28), Simone Muenst (23), Riccardo Murri (36), Charlotte KY Ng (23,32), Stefan Nicolet (23), Patrick GA Pedrioli (2), Lucas Pelkmans (35), Salvatore Piscuoglio (16,23), Michael Prummer (8,15), Mathilde Ritter (16), Christian Rommel (12), María L Rosano-González (8,15), Gunnar Rätsch (3,6,15,25), Jacobo Sarabia del Castillo (35), Ramona Schlenker (13), Petra C Schwalie (12), Severin Schwan (10), Tobias Schär (4), Gabriela Senti (26), Franziska Singer (8,15), Berend Snijder (2), Bettina Sobottka (28), Vipin T Sreedharan (8,15), Stefan Stark (3,6,15,25), Daniel J Stekhoven (8,15), Tinu M Thomas (3,6,15,25), Markus Tolnay (23), Nora C Toussaint (8,15), Mustafa A Tuncel (4), Audrey Van Drogen (7), Marcus Vetter (18), Tatjana Vlajnic (23), Sandra Weber (26), Walter P Weber (17), Rebekka Wegmann (2), Michael Weller (31), Fabian Wendt (7), Norbert Wey (28), Andreas Wicki (1,16,20), Bernd Wollscheid (7), Shuqing Yu (8,15), Johanna Ziegler (27), Marc Zimmermann (3,6,15,25), Martin Zoche (28), Gregor Zuend (30) (1) Cantonal Hospital Baselland, Medical University Clinic, Rheinstrasse 26, 4410 Liestal, Switzerland, (2) ETH Zurich, Department of Biology, Otto-Stern-Weg 3, 8093 Zurich, Switzerland, (3) ETH Zurich, Department of Biology, Wolfgang-Pauli-Strasse 27, 8093 Zurich, Switzerland, (4) ETH Zurich, Department of Biosystems Science and Engineering, Mattenstrasse 26, 4058 Basel, Switzerland, (5) ETH Zurich, Department of Chemistry and Applied Biosciences, Vladimir-Prelog-Weg 1-5/10, 8093 Zurich, Switzerland, (6) ETH Zurich, Department of Computer Science, Institute of Machine Learning, Universitätstrasse 6, 8092 Zurich, Switzerland, (7) ETH Zurich, Department of Health Sciences and Technology, Otto-Stern-Weg 3, 8093 Zurich, Switzerland, (8) ETH Zurich, NEXUS Personalized Health Technologies, John-von-Neumann-Weg 9, 8093 Zurich, Switzerland, (9) F. Hoffmann-La Roche Ltd, Grenzacherstrasse 124, 4070 Basel, Switzerland, (10) F. Hoffmann-La Roche Ltd, Grenzacherstrasse 124, 4070 Basel, Switzerland, , (11) Roche Diagnostics GmbH, Nonnenwald 2, 82377 Penzberg, Germany, (12) Roche Pharmaceutical Research and Early Development, Roche Innovation Center Basel, Grenzacherstrasse 124, 4070 Basel, Switzerland, (13) Roche Pharmaceutical Research and Early Development, Roche Innovation Center Munich, Roche Diagnostics GmbH, Nonnenwald 2, 82377 Penzberg, Germany, (14) Roche Pharmaceutical Research and Early Development, Roche Innovation Center Zurich, Wagistrasse 10, 8952 Schlieren, Switzerland, (15) Swiss Institute of Bioinformatics, Zurich, Switzerland, (16) University Hospital Basel and University of Basel, Department of Biomedicine, Hebelstrasse 20, 4031 Basel, Switzerland, (17) University Hospital Basel and University of Basel, Department of Surgery, Brustzentrum, Spitalstrasse 21, 4031 Basel, Switzerland, (18) University Hospital Basel, Brustzentrum & Tumorzentrum, Petersgraben 4, 4031 Basel, Switzerland, (19) University Hospital Basel, Brustzentrum, Spitalstrasse 21, 4031 Basel, Switzerland, (20) University Hospital Basel, Centre for Neuroendocrine & Endocrine Tumours, Spitalstrasse 21/Petersgraben 4, 4031 Basel, Switzerland, (21) University Hospital Basel, Department of Information- and Communication Technology, Spitalstrasse 26, 4031 Basel, Switzerland, (22) University Hospital Basel, Gynecological Cancer Center, Spitalstrasse 21, 4031 Basel, Switzerland, (23) University Hospital Basel, Institute of Medical Genetics and Pathology, Schönbeinstrasse 40, 4031 Basel, Switzerland, (24) University Hospital Basel, Spitalstrasse 21/Petersgraben 4, 4031 Basel, Switzerland, (25) University Hospital Zurich, Biomedical Informatics, Schmelzbergstrasse 26, 8006 Zurich, Switzerland, (26) University Hospital Zurich, Clinical Trials Center, Rämistrasse 100, 8091 Zurich, Switzerland, (27) University Hospital Zurich, Department of Dermatology, Gloriastrasse 31, 8091 Zurich, Switzerland, (28) University Hospital Zurich, Department of Pathology and Molecular Pathology, Schmelzbergstrasse 12, 8091 Zurich, Switzerland, (29) University Hospital Zurich, Institute for Anesthesiology, Rämistrasse 100, 8091 Zurich, Switzerland, (30) University Hospital Zurich, Rämistrasse 100, 8091 Zurich, Switzerland, (31) University Hospital and University of Zurich, Department of Neurology, Frauenklinikstrasse 26, 8091 Zurich, Switzerland, (32) University of Bern, Department of BioMedical Research, Murtenstrasse 35, 3008 Bern, Switzerland, (33) University of Zurich, Department of Quantitative Biomedicine, Winterthurerstrasse 190, 8057 Zurich, Switzerland, (34) University of Zurich, Grid Computing Competence Center, Rämistrasse 71, 8006 Zurich, Switzerland, (35) University of Zurich, Institute of Molecular Life Sciences, Winterthurerstrasse 190, 8057 Zurich, Switzerland, (36) University of Zurich, Services and Support for Science IT, Winterthurerstrasse 190, 8057 Zurich, Switzerland
